# 3D-QSAR study for the development of chalcone-based inhibitors targeting ovarian cancer cells with experimental validation

**DOI:** 10.3389/fphar.2026.1746658

**Published:** 2026-03-09

**Authors:** Manuel Valenzuela-Valderrama, Aranxa Varas, Mariaignacia Rubilar, Marcos Lorca, Jaime Mella, Christian Espinosa-Bustos, Marco Mellado, Javier Echeverría

**Affiliations:** 1 Laboratorio de Carcinogénesis Molecular, Facultad de Medicina y Ciencias de la Salud, Universidad Central de Chile, Santiago, Chile; 2 Carrera de Tecnología Médica, Facultad de Medicina y Ciencias de la Salud, Universidad Central de Chile, Santiago, Chile; 3 Facultad de Ciencias de la Vida, Carrera de Química y Farmacia, Universidad Viña del Mar, Viña del Mar, Chile; 4 Instituto de Química, Facultad de Ciencias, Universidad de Valparaíso, Valparaíso, Chile; 5 Centro de Investigación, Desarrollo e Innovación de Productos Bioactivos (CInBIO), Universidad de Valparaíso, Valparaíso, Chile; 6 Departamento de Farmacia, Facultad de Química y de Farmacia, Pontificia Universidad Católica de Chile, Santiago, Chile; 7 Dirección de Investigación, Universidad Bernardo O’Higgins, Santiago, Chile; 8 Centro de Investigación en Ingeniería de Materiales, Universidad Central de Chile, Santiago, Chile; 9 Departamento de Ciencias del Ambiente, Facultad de Química y Biología, Universidad de Santiago de Chile, Santiago, Chile

**Keywords:** 3D-QSAR, A2780 cell line, drug resistance, experimental validation, ovarian cancer, ROS

## Abstract

**Background:**

Ovarian cancer remains one of the most lethal gynecological malignancies, mainly due to late-stage diagnosis and frequent chemoresistance.

**Purpose:**

This study sought to develop 3D-QSAR models—Comparative Molecular Field and Similarity Index Analysis (CoMFA and CoMSIA)—to predict the antiproliferative activity of synthetic chalcone derivatives against A2780 ovarian cancer cells and to explore potential mechanisms of action through antioxidant response biomarkers.

**Materials and Methods:**

CoMFA and CoMSIA models were developed using a dataset of 64 chalcone derivatives and validated using q^2^, r^2^_ncv_, and other statistical metrics. Twelve chalcones predicted as active were synthesized and characterized by FT-IR and NMR spectroscopy. Their antiproliferative effects were evaluated using MTT assays, complemented by clonogenic testing, intracellular glutathione quantification, and analysis of biomarkers, including nuclear factor erythroid 2-related factor 2 (Nrf2), heme oxygenase-1 (HO-1), and NAD(P)H: quinone oxidoreductase 1 (NQO1). The most active compounds were further assessed in a cisplatin-resistant A2780 subline, with *N*-acetylcysteine (NAC) used to probe reactive oxygen species (ROS)-dependent mechanisms.

**Results and Discussion:**

The CoMFA and CoMSIA models demonstrated strong predictive performance (q^2^ = 0.763/0.789; r^2^_ncv_ = 0.963/0.920). Contour maps highlighted steric and electrostatic features linked to enhanced antiproliferative activity. The twelve synthesized chalcones exhibited experimental pIC_50_ values that strongly correlated with model predictions. Compounds **065**, **066**, and **072** showed the highest potency, with compound **072** also reducing clonogenic survival. Active derivatives increased intracellular glutathione and upregulated HO-1 without activating canonical Nrf2 signaling. In cisplatin-resistant A2780 cells, compounds **072** and **074** displayed markedly higher potency (IC_50_ = 6.50 and 10.22 μM) than cisplatin (93.4 μM). Their cytotoxicity was abrogated by NAC, indicating a ROS-dependent mode of action.

**Conclusion:**

CoMFA and CoMSIA models accurately predicted the activity of synthetic chalcones, and the biological findings identify these derivatives as promising candidates for the treatment of ovarian cancer, including chemoresistant forms.

## 1 Introduction

**FIGURE 1:**
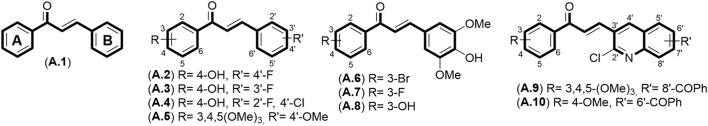
Chalcone structure (**A.1**) and related compounds (**A.2**–**A.10**) with inhibiting growth activity on ovarian cancer cell line.

Ovarian cancer is one of the most lethal malignancies affecting women worldwide, primarily due to its diagnosis at advanced stages of the disease (Momenimovahed et al., 2019). Although it ranks eighteenth in overall cancer incidence, it is the eighth most common cancer among women specifically (Ovarian cancer Statics, 2025). Its clinical management relies on cytoreductive surgery followed by chemotherapy; however, chemoresistance frequently emerges, substantially reducing treatment efficacy and contributing to high mortality rates (Shin et al., 2014; Wang et al., 2024). Although this standard approach achieves high initial response rates, approximately 70% of patients relapse, and many will eventually develop platinum-resistant disease, which is associated with low response rates to further chemotherapy and a median survival of about 1 year (Marchetti et al., 2019). These factors underscore the need for new therapeutic strategies and the development of novel chemotherapeutic agents capable of overcoming resistance and improving patient outcomes. Within this framework, chalcones (**A.1**) (Figure 1) have gained considerable interest as highly versatile scaffolds with documented antiproliferative properties across multiple cancer types, including breast (Kuete et al., 2015; Elkhalifa et al., 2020; Elkanzi et al., 2022), pancreatic (Padhye et al., 2010), colorectal (Takac et al., 2018; Jin et al., 2019), neuronal (Nishimura et al., 2007), leukemia (Pawlak et al., 2020), gastric (Zhang et al., 2016), and oral squamous carcinoma cells (Yamali et al., 2016). Their biological relevance stems from their simple yet chemically tunable α,β-unsaturated carbonyl system, which facilitates interactions with diverse molecular targets associated with cancer progression. For instance, the α,β-unsaturated carbonyl group behaves as a mild Michael acceptor, capable of interacting with nucleophilic cysteine residues in regulatory proteins. Through this electrophilic reactivity, chalcones modulate signaling pathways such as NF-κB, STAT3, PI3K/Akt, and MAPK, leading to decreased expression of prosurvival proteins (*e.g*., Bcl-2, Bcl-xL, survivin, and cyclin D1) and increased expression of proapoptotic proteins, including Bax and death receptors (Leon-Gonzalez et al., 2015; Rudrapal et al., 2021; Liu et al., 2022; Michalkova et al., 2023; Adhikari et al., 2025).

Importantly, synthetic chalcones (**A.2**–**A.4**) (Figure 1) have demonstrated potent growth-inhibitory activity against the A2780 ovarian cancer cell line, with effects comparable to those of paclitaxel (Sirka et al., 2022). Tetramethoxychalcone (**A.5**) (Figure 1) has demonstrated cytotoxicity in both drug-sensitive and cisplatin-resistant ovarian cancer lines, among them A2780, A2780/CDDP, and SKOV-3, through mechanisms involving modulation of the cell cycle and induction of apoptosis, including the downregulation of cyclin D1 and CDK4 and the upregulation of p16, p21, and p27, together with the activation of pro-apoptotic signaling (Qi et al., 2014). Semi-synthetic chalcones derived from syringaldehyde (**A.6**–**A.8**) (Figure 1) also exhibit notable cytotoxicity, with IC_50_ values below 10 μM (Pan et al., 2024). In addition, hybrid chalcone-based molecules such as quinoline–chalcone derivatives (**A.9**–**A.10**) (Figure 1) have shown promising activity, with compound **A.9** inhibiting tubulin polymerization in A2780 cells (Mirzaei et al., 2020).

The antiproliferative effects of chalcones are associated with several mechanisms, including modulation of signaling pathways governing apoptosis (e.g., PI3K/AKT and MAPK) (Takac et al., 2018; Li et al., 2019; Michalkova et al., 2022; Jin et al., 2023), induction of oxidative stress, cell-cycle arrest (Salanci et al., 2024), inhibition of tubulin polymerization (Mirzaei et al., 2020), and interaction with key regulatory enzymes. Despite these advantages, chalcone-based anticancer leads still face significant limitations, such as variable potency, limited selectivity, moderate solubility, and susceptibility to metabolic degradation (Rautio et al., 2008; Adhikari et al., 2025). Consequently, rational structural optimization is essential to enhance their pharmacological profiles and to identify new derivatives with effective activity against ovarian cancer, including drug-resistant phenotypes.

To address these challenges, computational methodologies have become indispensable for accelerating drug discovery efforts. Three-dimensional quantitative structure–activity relationship (3D-QSAR) modeling—particularly Comparative Molecular Field Analysis (CoMFA) and Comparative Molecular Similarity Indices Analysis (CoMSIA)—provides detailed insights into steric and electrostatic features that modulate biological activity (Cramer et al., 1988; Klebe, 2000; Sliwoski et al., 2014; Ouma et al., 2024). These approaches enable the identification of key structural determinants that guide the rational design of novel chalcone derivatives. However, to achieve practical relevance, computational predictions must be validated experimentally (Sliwoski et al., 2014).

In this context, the present work was designed to address these gaps by combining 3D-QSAR modeling with experimental validation and mechanistic investigation in human ovarian cancer cells. We first compiled a structurally diverse set of chalcones with reported cytotoxicity against A2780 cells and developed CoMFA and CoMSIA models to define the steric, electrostatic, and hydrophobic requirements for antiproliferative activity. Guided by these contour maps, we then designed and synthesized a focused series of new chalcones that emphasize favorable steric and electrostatic features while retaining the Michael acceptor motif. The antiproliferative and clonogenic effects of these compounds were evaluated in both cisplatin-sensitive A2780 cells and their cisplatin-resistant counterpart, A2780-CispR. In parallel, we characterized their impact on cellular redox status by measuring intracellular ROS, total glutathione, and key components of the antioxidant response, including Nrf2, HO-1, NQO1, glutathione reductase, and γ-glutamylcysteine synthetase. The use of *N*-acetylcysteine (NAC) as a thiol antioxidant allowed us to test the functional contribution of ROS and electrophilic signaling to the antiproliferative effects.

By integrating ligand-based 3D-QSAR with targeted synthesis, cellular phenotypic assays, and redox-focused mechanistic studies in both platinum-sensitive and -resistant ovarian cancer models, this study provides a coherent rationale for the design of chalcone derivatives as antiproliferative agents. The resulting models identify structural determinants that can be exploited to enhance activity and, potentially, selectivity, while the mechanistic data delineate how these synthetic chalcones engage ROS-dependent pathways and the Nrf2–HO-1 axis in ovarian cancer cells. Together, these findings contribute to a more informed framework for optimizing chalcone-based scaffolds as candidates for further preclinical development in ovarian cancer.

## 2 Materials and methods

### 2.1 Theoretical models

#### 2.1.1 Three-dimensional quantitative structure-activity relationship model

The three-dimensional quantitative structure–activity relationship (3D-QSAR) models were developed using Sybyl-X software, version 1.2 (Tripos Inc., St. Louis, MO, USA), employing Comparative Molecular Field Analysis (CoMFA) and Comparative Molecular Similarity Indices Analysis (CoMSIA), as in our previous studies (Luczywo et al., 2023). The reported biological activities, expressed as IC_50_, were converted into negative logarithmic values (pIC_50_ = –log_10_(IC_50_)) in mol/L.

Each compound was initially drawn in ChemDraw (PerkinElmer Inc., Shelton, CT, USA) and then energy-minimized using the Tripos force field. Gasteiger–Hückel charges were then calculated for each atom. Molecular alignment was performed atom-by-atom using the α,β-unsaturated carbonyl fragment as the common structural reference (Supplementary Figure S1).

#### 2.1.2 Dataset selection and inhibitory activity

The dataset was compiled from studies published by the research groups of Ghodsi, Basaveswara Rao, Koran, Yang, Prameela Subhashini, and Zhao (Qi et al., 2014; Bujji et al., 2020; Mirzaei et al., 2020; Srilaxmi et al., 2021; Sirka et al., 2022; Pan et al., 2024), based on consistency in cell culture conditions and the antiproliferative assay method (MTT). A total of 64 bioactive compounds were identified and included in the analysis (Supplementary Table S1). These compounds were randomly divided into a training set (44 compounds, 69%) and a test set (20 compounds, 31%). The distributions of pIC_50_ values for the entire dataset and the training and test sets are shown in Supplementary Figure S2.

#### 2.1.3 Comparative molecular field analysis (CoMFA)

To derive the CoMFA descriptor fields, the aligned molecules from the training set were positioned within a three-dimensional cubic lattice with a 2 Å grid spacing in the x, y, and z directions, ensuring complete coverage of all compounds. Steric and electrostatic field energies were calculated using a sp^3^ carbon probe atom with a van der Waals radius of 1.52 Å and a charge of +1.0. Cutoff values for both steric and electrostatic fields were set at 30.0 kcal/mol.

#### 2.1.4 Comparative molecular similarity index analysis (CoMSIA)

For the CoMSIA analysis, standard settings were applied, including a probe with a charge of +1.0, a radius of 1 Å, and hydrophobicity, hydrogen-bond-donating capability, and hydrogen-bond-accepting capability, each set to +1.0 (Klebe et al., 1994). Five different fields were calculated: steric, electrostatic, hydrophobic, hydrogen-bond acceptor, and hydrogen-bond donor. A Gaussian-type distance dependence was employed to evaluate the relative attenuation of the field at each atom’s position within the lattice. The attenuation factor (α) was set to the default value of 0.3.

#### 2.1.5 Internal validation and partial least squares (PLS) analysis

Partial least squares (PLS) analysis was employed to establish a linear correlation between the CoMSIA descriptors (independent variables) and the biological activity values (dependent variables) (Clark et al., 1989). To identify the optimal model, cross-validation was performed using the leave-one-out (LOO) method combined with sample-distance partial least squares (SAMPLS), which yields the cross-validation coefficient of determination (q^2^) and determines the optimal number of components (N). A non-cross-validated analysis was subsequently conducted with a column filter value of 2.0 to accelerate computation and minimize noise. The q^2^, serving as a measure of the internal predictive quality of the model, was calculated according to Equation 1:

$$q^{2}=1-\frac{\sum \left(y_{i}-y_{pred}\right)}{\sum \left(y_{i}-y_{ave}\right)}$$ (1)

where y_i_ is the observed activity of the training set, y_pred_ is the predicted activity of the training set, and y_ave_ is the average activity of the training set.

#### 2.1.6 External validation of the CoMSIA model

The statistical evaluation of the QSAR models was conducted using several performance metrics, calculated by a custom Python script executed in JupyterLab. The dataset, stored in a single CSV file containing both training and test sets, was imported using the pandas library. The coefficient of determination (r^2^) was computed to assess the proportion of variance in experimental values explained by the model predictions. In contrast, the concordance correlation coefficient (CCC) quantified both precision and accuracy. Three versions of the predictive squared correlation coefficient were determined: q^2^F1, calculated relative to the mean of experimental values; q^2^F2, using the mean of predicted values; and q^2^F3, adjusted for intercept deviations from the experimental mean. Mean Absolute Error (MAE) and Root Mean Square Deviation (RMSD) measure the average and squared differences between predicted and observed activities, respectively. The r^2^_0_ metric was obtained from a linear regression forced through the origin, whereas the metric r^2^ (r^2^_m_) penalized discrepancies between r^2^ and r^2^_0_ to detect systematic prediction bias. Finally, Δr^2^_m_ was computed as the absolute difference between r^2^_m_ values obtained from direct and inverse regressions, thereby assessing model robustness and symmetry. All calculations were performed automatically on both the training and test sets, and the results were exported to a CSV file for further analysis.

#### 2.1.7 *Y*-randomization test

To ensure that the CoMFA and CoMSIA models were not the result of chance correlation, the activity values (pIC_50_) were randomly shuffled over ten iterations, and the statistical parameters q^2^ and r^2^ (non-cross-validated) were recalculated for each iteration. The absence of chance correlation was confirmed by observing low q^2^ and/or r^2^ values across all randomized iterations.

#### 2.1.8 Applicability domain calculation

To assess the structural applicability domain of the QSAR model, the Tanimoto similarity index was calculated for each compound in the studied series (Bajusz et al., 2015). Molecular structures were encoded as Simplified-Molecular-Input-Line-Entry System (SMILES) and converted into molecular fingerprints using the Morgan algorithm (radius = 2, 1,024 bits) implemented in RDKit. For each molecule, the maximum Tanimoto similarity (Tanimoto_max) was determined by comparing it to all other molecules in the dataset, excluding self-comparisons. A threshold of 0.60 was applied to classify compounds as structurally within the model’s applicability domain (Tanimoto_max ≥ 0.60) or as structural outliers (Tanimoto_max < 0.60). Furthermore, residuals were calculated as the difference between the experimental and predicted pIC_50_ values for each compound. A scatter plot of Tanimoto_max versus residuals was generated to identify compounds that were structural outliers, predictive outliers (absolute residual > 1.0 log units), or both.

### 2.2 Chemistry

#### 2.2.1 Instrumentation

FT-IR spectra were recorded on a Jasco FT/IR-4X with ATR (Tokyo, Japan). Spectra were collected from 4,000 to 500 cm^-1^; samples were analyzed in the solid-state using ATR. ^1^H and ^13^C NMR spectra were recorded on a Bruker Avance 400 digital or Bruker Avance 200 NMR spectrometers (Berlin, Germany), operating at 400 or 200 MHz for ^1^H and 100 MHz or 50 MHz for ^13^C, respectively.

#### 2.2.2 General procedure to obtain chalcones (065–076)

Compounds **067**–**072** were synthesized in alkaline media following a previously reported procedure (Jeon et al., 2012). In a dry 100-mL round-bottom flask, commercial acetophenone (**I**, 250 mg, 2.08 mmol) and the respective substituted benzaldehyde (**II**, 2.50 mmol, corresponding to 1.2 mol equivalents) were combined. Both reagents were dissolved in ethanol (10 mL), after which a saturated NaOH solution was added dropwise. The reaction mixture was stirred for 48 h, and then quenched by adding 5% HCl until the pH reached approximately 7. The mixture was subsequently extracted with dichloromethane (CH_2_Cl_2_, 3 × 20 mL). The combined organic layers were dried over anhydrous Na_2_SO_4_, filtered, and purified by column chromatography using a hexane/ethyl acetate (EtOAc) gradient as the eluent. The gradual increase in polarity facilitated the isolation of compounds **067**–**072**, yielding 55%–99%. The FT-IR, ^1^H-, and ^13^C-NMR spectra are in the Supplementary Material as Spectrum S1-S36.

Compounds **065**–**066** and **073**–**076** were synthesized under acidic conditions using concentrated sulfuric acid, according to a previously reported protocol (Janse van Rensburg et al., 2021). In a dry 100-mL round-bottom flask, acetophenone (**I**, 250 mg, 2.08 mmol) and the corresponding substituted benzaldehyde (**II**, 2.50 mmol, 1.2 equivalents) were dissolved in ethanol (10 mL). Concentrated sulfuric acid (5 mL) was then added dropwise, and the mixture was stirred for 48 h. The reaction was quenched by adding 20 mL of water, yielding a solid precipitate. The solid was filtered and dried. The mixture was then extracted with dichloromethane (CH_2_Cl_2_, 3 mL × 20 mL). The combined organic phase was dried over anhydrous Na_2_SO_4_, filtered, and purified by column chromatography using a hexane/EtOAc solvent system. This polarity gradient enabled the isolation of the desired compounds in yields ranging from 65% to 89%.

(*E*)-3-(3-hydroxyphenyl)-1-phenylprop-2-en-1-one **(065)**: Pale brown solid. Yield. 65%. Mp 220 °C–224 °C. FT-IR (cm^-1^): υ 3,320 (OH), 1,648 (CO), 1,600 (CH), 1,570 (CH), 1,489 (CH), 1,234 (COH), 1,215 (COH). ^1^H-NMR (200 MHz, Acetone-d_6_, δ, ppm): 8.70 (1H, s br, OH), 8.14 (2H, dd, *J* = 8.2 Hz, 1.5 Hz, CH-2 + CH-6), 7.75 (2H, d, *J* = 1.5 Hz, CH-3 + CH-5), 7.59 (1H, d, *J* = 14.0 Hz, CH-β), 7.70–7.46 (1H, m, CH-4), 7.56 (1H, d, *J* = 14.0 Hz, CH-α), 7.29 (3H, m, CH-2’ + CH-4′ + CH-6′), 7.01–6.90 (1H, m, CH-5′). ^13^C-NMR (50 MHz, Acetone-d_6_, δ, ppm): 190.1 (C=O), 158.8 (C), 145.0 (CH), 139.1 (C), 137.3 (C), 133.7 (CH), 130.8 (CH), 129.5 (2xCH), 129.3 (2xCH), 122.9 (CH), 121.0 (CH), 118.6 (CH), 115.9 (CH). All spectroscopic data are concordant with a previous report (Ren et al., 2017).

(*E*)-3-(4-hydroxyphenyl)-1-phenylprop-2-en-1-one **(066)**: Pale brown solid. Yield: 85%. Mp 183 °C–187 °C. FT-IR (cm^-1^): υ 3,204 (OH), 1,647 (CO), 1,598 (CH), 1,579 (CH), 1,510 (CH), 1,216 (COH). ^1^H-NMR (400 MHz, Acetone-d_6_, δ, ppm): 8.11 (2H, d, *J* = 8.4 Hz, CH-2 + CH-6), 7.75 (1H, d, *J* = 15.6 Hz, CH-β), 7.71 (2H, d, *J* = 8.9 Hz, CH-2′ + CH-6′), 7.61 (1H, t, *J* = 8.4 Hz, CH-4), 7.54 (2H, d, *J* = 8.4 Hz, CH-3 + CH-5), 7.53 (1H, d, *J* = 15.6 Hz, CH-α), 6.92 (2H, d, *J* = 8.9 Hz, CH-3′ + CH-5′). ^13^C-NMR (100 MHz, Acetone-d_6_, δ, ppm): 189.9 (C=O), 160.8 (C), 145.2 (CH), 139.4 (C), 133.3 (CH), 131.5 (2xCH), 129.4 (2xCH), 129.1 (2xCH), 127.5 (C), 119.6 (CH), 116.7 (2xCH). All spectroscopic data are concordant with a previous report (Mellado et al., 2022).

(*E*)-3-(3-methoxyphenyl)-1-phenylprop-2-en-1-one **(067)**: Pale yellow solid. Yield: 55%. Mp 60 °C–62 °C. FT-IR (cm^-1^): υ 2,937 (CH), 1,660 (CO), 1,605 (CH), 1,594 (CH), 1,577 (CH), 1,493 (CH), 1,252 (COC), 1,171 (COC). ^1^H-NMR (400 MHz, CDCl_3_, δ, ppm): 8.01 (2H, d, *J* = 6.8 Hz, CH-2 + CH-6), 7.76 (1H, d, *J* = 15.7 Hz, CH-β), 7.48 (2H, d, *J* = 6.8 Hz, CH-3 + CH-5), 7.47 (1H, m, CH-4), 7.46 (1H, d, *J* = 15.7 Hz, CH-α), 7.30 (1H, t, *J* = 7.8 Hz, CH-5′), 7.23 (1H, dd, *J* = 7.8 Hz, 2.5 Hz, CH-6′), 7.14 (1H, d, *J* = 2.5 Hz, H2′), 6.94 (1H, ddd, *J* = 7.8 Hz, 2.5 Hz, 2.5 Hz, CH-4′), 3.80 (3H, s, CH_3_O-C-3′). ^13^C-NMR (100 Hz, CDCl_3_, δ, ppm): 190.2 (C=O), 159.7 (C), 144.5 (CH), 137.9 (C), 136.0 (C), 132.6 (CH), 129.8 (2xCH), 128.4 (2xCH), 121.9 (CH), 120.9 (CH), 116.1 (CH), 113.3 (2xCH), 55.2 (CH_3_O). All spectroscopic data are concordant with a previous report (Mellado et al., 2022).

(*E*)-3-(4-methoxyphenyl)-1-phenylprop-2-en-1-one **(068)**: Pale orange solid. Yield: 99%. Mp 70 °C–72 °C. FT-IR (cm^-1^): υ 2,955 (CH), 1,656 (CO), 1,595 (CH), 1,577 (CH), 1,510 (CH), 1,261 (COC), 1,211 (COC). ^1^H-NMR (400 MHz, CDCl_3_, δ, ppm): 8.01 (2H, d, *J* = 7.5 Hz, CH-2 + CH-6), 7.89 (1H, d, *J* = 15.6 Hz, CH-β), 7.60 (2H, d, *J* = 8.7 Hz, CH-2′ + CH-6′), 7.57 (1H, t, *J* = 7.5 Hz, CH-4), 7.49 (2H, d, *J* = 7.5 Hz, CH-3 + CH-5), 7.42 (1H, d, *J* = 15.6 Hz, CH-α), 6.93 (2H, d, *J* = 8.7 Hz, CH-3′ + CH-5′), 3.85 (3H, s, CH_3_O-C-4′). ^13^C-NMR (100 MHz, CDCl_3_, δ, ppm): 190.5 (C=O), 161.6 (C), 144.7 (C), 138.4 (C), 132.5 (C), 130.2 (2xCH), 128.5 (2xCH), 128.4 (2xCH), 127.5 (C), 119.7 (Cα), 114.4 (2xCH), 55.4 (CH_3_O). All spectroscopic data are concordant with a previous report (Bustos et al., 2022).

(*E*)-3-(4-hydroxy-3-methoxyphenyl)-1-phenylprop-2-en-1-one (**069**): Pale orange solid. Yield: 81%. Mp 81 °C–83 °C. FT-IR (cm^-1^): υ 3,314 (OH), 1,654 (CO), 1,581 (CH), 1,560 (CH), 1,510 (CH), 1,243 (COH), 1,210 (COC), 1,168 (COH), 1,120 (COC). ^1^H-NMR (400 MHz, CDCl_3_, δ, ppm): 8.01 (2H, d, *J* = 7.5 Hz, CH-2 + CH-6), 7.75 (1H, d, *J* = 15.6 Hz, CH-β), 7.57 (1H, d, *J* = 7.1 Hz, CH-4), 7.50 (2H, t, *J* = 7.5 Hz, CH-3 + CH-5), 7.38 (1H, d, *J* = 15.6 Hz, CH-α), 7.22 (1H, dd, *J* = 8.3 Hz, 1.5 Hz, CH-6′), 7.13 (1H, *J* = 1.5 Hz, CH-2′), 6.96 (1H, d, *J* = 8.3 Hz, CH-5′), 3.96 (3H, s, CH_3_O-C-3′). ^13^C-NMR (100 MHz, CDCl_3_, δ, ppm): 190.7 (C), 148.3 (C), 146.8 (C), 145.3 (CH), 138.4 (C), 132.6 (CH), 128.5 (2xCH), 128.4 (2xCH), 127.4 (C), 123.4 (CH), 119.7 (CH), 114.9 (CH), 110.0 (CH), 56.0 (CH_3_O). All spectroscopic data are concordant with a previous report (González-Vergara et al., 2022).

(*E*)-3-(3,4-dimethoxyphenyl)-1-phenylprop-2-en-1-one (**070**): Orange oil. Yield: 79%. FT-IR (cm^-1^): υ 2,940 (CH), 1,653 (CO), 1,584 (CH), 1,572 (CH), 1,510 (CH), 1,252 (COC), 1,236 (COC), 1,163 (COC), 1,130 (COC). ^1^H-NMR (400 MHz, CDCl_3_, δ, ppm): 7.96 (2H, d, *J* = 7.7 Hz, CH-2 + CH-6), 7.71 (1H, d, *J* = 15.6 Hz, CH-β), 7.50 (1H, t, *J* = 7.7 Hz, CH-4), 7.42 (2H, d, *J* = 7.5 Hz, CH-3 + CH-5), 7.35 (1H, d, *J* = 15.6 Hz, CH-α), 7.16 (1H, d, *J* = 8.3 Hz, CH-6′), 7.11 (1H, s, CH-2′), 6.82 (1H, d, *J* = 8.3 Hz, CH-5′), 3.87 (3H, s, CH_3_O-C-4′), 3.84 (3H, s, CH_3_O-C-3′). ^13^C-NMR (100 MHz, CDCl_3_, δ, ppm): 190.2 (C=O), 151.2 (C), 149.0 (C), 144.7 (CH), 138.2 (C), 132.3 (CH), 128.3 (2xCH), 128.1 (2xCH), 127.6 (C), 123.0 (CH), 119.7 (CH), 110.9 (CH), 109.9 (CH), 55.7 (2xCH_3_O). All spectroscopic data are concordant with a previous report (Mellado et al., 2018).

(*E*)-3-(benzo[*d*][1,3]dioxol-5-yl)-1-phenylprop-2-en-1-one (**071**): Yellow solid. Yield: 95%. Mp 48 °C–50 °C. FT-IR (cm^-1^): υ 2,914 (CH), 1,660 (CO), 1,595 (CH), 1,590 (CH), 1,575 (CH), 1,244 (COC), 1,214 (COC). ^1^H-NMR (400 MHz, CDCl_3_, δ, ppm): 8.00 (2H, d, *J* = 7.6 Hz, CH-2 + CH-6), 7.73 (1H, d, *J* = 15.6 Hz, CH-β), 7.57 (1H, t, *J* = 7.6 Hz, CH-4), 7.48 (2H, d, *J* = 7.6 Hz, CH-3 + CH-5), 7.36 (1H, d, *J* = 15.6 Hz, CH-α), 7.16 (1H, s, CH-2′), 7.11 (1H, d, *J* = 8.0 Hz, H6′), 6.83 (1H, d, *J* = 8.0 Hz, H5′), 6.00 (2H, s, OCH_2_O). ^13^C-NMR (100 MHz, CDCl_3_, δ, ppm): 190.2 (C=O), 149.8 (C), 148.3 (C), 144.6 (CH), 138.3 (C), 132.5 (C), 129.2 (C), 128.5 (2xCH), 128.3 (2xCH), 125.1 (CH), 120.0 (CH), 108.6 (CH), 106.6 (CH), 101.5 (OCH_2_O). All spectroscopic data are concordant with a previous report (Mellado et al., 2020).

(*E*)-1-phenyl-3-(3,4,5-trimethoxyphenyl)prop-2-en-1-one **(072)**: Pale orange solid. Yield: 92%. Mp 134 °C–137 °C. FT-IR (cm^-1^): υ 2,940 (CH), 1,660 (CO), 1,599 (CH), 1,586 (CH), 1,576 (CH), 1,248 (COC), 1,214 (COC), 1,125 (COC). ^1^H-NMR (300 MHz, CDCl_3_, δ, ppm): 8.02 (2H, d, *J* = 7.2 Hz, CH-2 + CH-6), 7.73 (1H, d, *J* = 15.6 Hz, CH-β), 7.59 (1H, t, *J* = 7.2 Hz, CH-4), 7.51 (2H, d, *J* = 7.2 Hz, CH-3 + CH-5), 7.42 (1H, d, *J* = 15.6 Hz, CH-α), 6.87 (2H, s, CH-2’ + CH-6′), 3.92 (6H, s, CH_3_O-C-3’ + CH_3_O-C-5′), 3.90 (3H, s, CH_3_O-C-4′).^13^C-NMR (75 MHz, CDCl_3_, δ, ppm): 190.6 (C=O), 153.5 (2xC), 145.1 (CH), 140.3 (C), 138.2 (C), 132.8 (CH), 130.4 (CH), 128.7 (2xCH), 128.5 (2xCH), 121.4 (C), 105.5 (2xCH), 61.0 (CH_3_O), 56.2 (2xCH_3_O). All spectroscopic data are concordant with a previous report (Ducki et al., 2009).

(*E*)-3-(4-fluorophenyl)-1-phenylprop-2-en-1-one **(073)**: Pale yellow solid. Yield: 78%. Mp. 81 °C–84 °C. FT-IR (cm^-1^): υ 2,924 (CH), 1,659 (CO), 1,602 (CH), 1,592 (CH), 1,586 (CH), 1,576 (CH), 1,446 (CF), 1,212 (CF). ^1^H-NMR (200 MHz, CDCl_3_, δ, ppm): 8.15 (2H, d, *J* = 6.7 Hz, CH-2 + CH-6), 7.98–7.86 (1H, m, CH-2’ + CH-6′), 7.81 (2H, d, *J* = 2.9 Hz, CH-3 + CH-5), 7.67–7.50 (1H, m, CH-4), 7.59 (1H, d, *J* = 14.2 Hz, CH-β), 7.56 (1H, d, *J* = 14.2 Hz, CH-α), 7.23 (2H, dt, *J* = 8.8 Hz, 2.1 Hz, CH-3′ + CH-5′). ^13^C-NMR (50 MHz, CDCl_3_, δ, ppm): 189.9 (C=O), 164.8 (d, *J* = 249.3 Hz C-F_J1_), 143.5 (CH), 139.1 (C), 133.7 (CH), 132.6 (d, *J* = 3.3 Hz, C-F_J4_), 131.8 (2xCH, d, *J* = 8.6 Hz, CH-F_J3_), 129.5 (2xCH), 129.3 (2xCH), 122.8 (CH, d, *J* = 2.4 Hz, CH-F_J4_), 116.7 (2xCH, d, *J* = 22.0 Hz, C-F_J2_). All spectroscopic data are concordant with a previous report (Jiang et al., 2015).

(*E*)-3-(3-chlorophenyl)-1-phenylprop-2-en-1-one (**074**): Pale yellow solid. Yield: 89%. Mp 68 °C–71 °C. FT-IR (cm^-1^): υ 2,932 (CH), 1,660 (CO), 1,605 (CH), 1,594 (CH), 1,579 (CH), 1,564 (CH), 1,448 (CH), 860 (CCl), 771 (CCl). ^1^H-NMR (200 MHz, CDCl_3_, δ, ppm): 8.17 (2H, dt, *J* = 6.8 Hz, 1.9 Hz, CH-2 + CH-6), 7.96 (1H, d, *J* = 15.7 Hz, CH-β), 7.92 (1H, s, CH-4), 7.77–7.74 (1H, m, CH-6′), 7.75 (1H, d, *J* = 15.7 Hz, CH-α), 7.65 (1H, dt, *J* = 3.1 Hz, 2.5 Hz, CH-2′), 7.58 (2H, dt, *J* = 6.8 Hz, 1.9 Hz, CH-3 + CH-5), 7.49–7.46 (2H, m, CH-4′ + CH-5′). ^13^C-NMR (50 MHz, CDCl_3_, δ, ppm): 189.8 (C=O), 143.0 (CH), 138.9 (C), 138.2 (C), 135.3 (C), 133.9 (CH), 131.4 (CH), 130.9 (CH), 129.6 (2xCH), 129.4 (2xCH), 128.8 (CH), 128.2 (CH), 124.4 (CH). All spectroscopic data are concordant with a previous report (Ludovico et al., 2021).

(*E*)-3-(4-chlorophenyl)-1-phenylprop-2-en-1-one (**075**): White solid. Yield: 84%. Mp 110 °C–114 °C. FT-IR (cm^-1^): υ 2,962 (CH), 1,658 (CO), 1,601 (CH), 1,590 (CH), 1,577 (CH), 1,565 (CH), 1,486 (CH), 820 (CCl), 773 (CCl). ^1^H-NMR (200 MHz, Acetone-d_6_, δ ppm): 8.89–7.83 (2H, m, CH-3 + CH-5), 8.15 (2H, dd, *J* = 8.2 Hz, 1.5 Hz, CH-2 + CH-6), 7.90 (1H, d, *J* = 15.7 Hz, CH-β), 7.84 (1H, d, *J* = 15.7 Hz, CH-α), 7.71–7.65 (1H, m, CH-4), 7.57 (2H, d, *J* = 7.4 Hz, CH-3’ + CH-5′), 7.49 (2H, d, *J* = 7.4 Hz, 5.0 Hz, CH-2′ + CH-6′). ^13^C-NMR (50 MHz, Acetone-d_6_, δ ppm): 189.9 (C=O), 143.3 (CH), 139.0 (C), 136.5 (C), 134.9 (C), 133.8 (CH), 131.1 (2xCH), 129.9 (2xCH), 129.6 (2xCH), 129.3 (2xCH), 123.6 (CH). All spectroscopic data are concordant with a previous report (Jiang et al., 2015).

(*E*)-3-(4-hydroxy-3-nitrophenyl)-1-phenylprop-2-en-1-one (**076**): Yellow solid. Yield: 66%. Mp. 134 °C–138 °C. FT-IR (cm^-1^): υ 3,195 (OH), 2,921 (CH), 1,657 (CO), 1,605 (CH), 1,575 (CH), 1,532 (NO_2_), 1,490 (CH), 1,488 (CH), 1,422 (COH) 1,303 (NO_2_), 1,173 (COH). ^1^H-NMR (200 MHz, Acetone-d_6_, δ ppm): 8.40 (1H, d, *J* = 2.2 Hz, CH-2′), 8.13–7.98 (3H, m, CH-2 + CH-4 + CH-6), 7.81 (1H, d, *J* = 15.7 Hz, CH-β), 7.66 (1H, d, *J* = 15.7 Hz, CH-α), 7.52–7.38 (3H, m, CH-3 + CH-5 + CH-6′), 7.15 (1H, d, *J* = 8.8 Hz, CH-5′). ^13^C-NMR (50 MHz, Acetone-d_6_, δ, ppm): 189.7 (C=O), 156.4 (C), 142.2 (CH), 138.9 (C), 137.2 (CH + C), 133.8 (CH), 129.6 (2xCH), 129.4 (2xCH), 128.7 (C), 126.5 (CH), 123.3 (CH), 121.4 (CH). All spectroscopic data are concordant with a previous report (Mellado et al., 2021).

### 2.3 Cytotoxic assays

#### 2.3.1 Cell culture

The human epithelial ovarian cancer cell line A2780 was obtained from the European Collection of Authenticated Cell Cultures (ECACC, Porton Down, United Kingdom). A2780 cells were cultured in Roswell Park Memorial Institute (RPMI) 1,640 medium, supplemented with 10% fetal bovine serum (GE Healthcare) and antibiotics (100 U/mL penicillin, 100 µg/mL streptomycin) in a humidified atmosphere with 5% CO_2_ at 37 °C. The A2780-Cisp^R^ cell line was obtained by exposing the parental A2780 cells to increasing concentrations of cisplatin (0.5–3 µM) over approximately 6 months, until a stable resistant line was established.

#### 2.3.2 Cell proliferation

The effect of chalcone treatment on A2780 cell proliferation was assessed using the MTT assay (Berridge et al., 2005). A2780 cells were treated with chalcone derivatives at concentrations ranging from 5 to 100 μM, using 0.1% dimethyl sulfoxide (DMSO) in supplemented RPMI 1640 medium. Cells exposed only to 0.1% DMSO in the same medium served as the solvent control. Additionally, the antiproliferative drug cisplatin was used as a positive control. After 24 h of incubation, cell viability was assessed by adding 100 µL of MTT solution (0.5 mg/mL in supplemented RPMI 1640) and incubating for 90 min. Subsequently, cells were lysed with 100 µL of solubilization solution and agitated for 2 h to release formazan. Absorbance was then measured at 570 nm using a TECAN Infinite M200 PRO plate reader (Tecan Trading AG, Switzerland). All conditions were tested in triplicate.

#### 2.3.3 Clonogenic assay

The clonogenic capacity of A2780 cells was evaluated as previously described (Carrasco et al., 2023). Briefly, cells were cultured for 24 h in complete RPMI-1640 medium, and then treated with selected chalcones (20 µM) for an additional 24 h. After treatment, cells were trypsinized, and cell viability was assessed using the trypan blue exclusion assay. Viable (trypan blue-negative) cells were reseeded at a density of 1,000 cells per well in 6-well plates and incubated for 7 days to allow colony formation. Colonies were then fixed with 100% methanol for 15 min at room temperature, stained with 0.5% crystal violet for 30 min, photographed, and counted.

#### 2.3.4 Western blot analysis

Protein extracts were prepared using a lysis buffer containing 1% Triton X-100 and 1X cOmplete™ Mini protease inhibitor cocktail (Merck-Roche, Darmstadt, Germany), as previously described (Carrasco et al., 2023). Protein concentrations were determined using the Pierce™ BCA Protein Assay Kit according to the manufacturer’s instructions (Thermo Fisher Scientific). Equal amounts of protein (80 μg per lane) were separated by sodium dodecyl sulfate-polyacrylamide gel electrophoresis (SDS–PAGE) using 10% mini-gels (Bio-Rad) and transferred to nitrocellulose membranes, as described previously (Carrasco et al., 2023). Membranes were blocked in PBS containing 5% skim milk and 0.1% Tween-20 (PBST) and then incubated with the appropriate primary antibodies. The following mouse monoclonal antibodies were obtained from Santa Cruz Biotechnology (Santa Cruz, CA, USA): anti-γ-GCSc (sc-166382), anti-HO-1 (sc-136960), and NQO-1 (sc-32793). Mouse monoclonal anti-β-Actin (A5316) was purchased from Merck-Sigma-Aldrich. Rabbit monoclonal anti-Nrf2 (33649) was purchased from Cell Signaling. The HRP-conjugated secondary antibody used was donkey anti-mouse (SA1-100; Thermo Fisher Scientific). Detection of HRP activity was performed using the EZ-ECL reagent (Biological Industries), and chemiluminescent signals were captured with an ImageQuant LAS500 imager (General Electric).

#### 2.3.5 GSH content

Total glutathione (GSH) content was measured based on the formation of the yellow-colored derivative 5′-thio-2-nitrobenzoic acid (TNB), produced during the reaction of GSH with the sulfhydryl reagent 5,5′-dithiobis(2-nitrobenzoic acid) (DTNB), and quantified spectrophotometrically at 412 nm, as previously described (Valenzuela et al., 2014).

#### 2.3.6 Statistical analysis

All data are presented as mean ± S.D. from at least three independent experiments. Statistical analyses were performed using Prism version 10.5.0 software (GraphPad, Boston, MA, USA). Differences were considered statistically significant at *p* < 0.05, as determined by one-way ANOVA followed by Dunnett’s or Tukey’s *post hoc* test.

## 3 Results and discussion

### 3.1 3D-QSAR models

The 3D-QSAR models (CoMFA and CoMSIA) were developed based on the structural diversity reported in the dataset (Supplementary Table S1), which comprises 64 compounds containing the chalcone pharmacophoric core and exhibiting growth-inhibitory activity against the A2780 ovarian cancer cell line. One key advantage of computational models, such as 3D-QSAR, is their ability to identify structural features that modulate cytotoxic effects—in this study, specifically in the A2780 cell line. This approach helps focus synthetic efforts, thereby reducing the high costs associated with laboratory work during lead compound discovery (Lorca et al., 2024).

The CoMFA and CoMSIA models were constructed using the 64-compound dataset, which was randomly split into a training set of 44 compounds (69%) and a test set of 20 compounds (31%), as shown in Supplementary Figure S2. The IC_50_ values were converted to pIC_50_ values (–log_10_(IC_50_), covering an activity range of 3.8 logarithmic units (pIC_50_ max = 7.921, pIC_50_ min = 4.089). To obtain the best CoMFA and CoMSIA models, a systematic search was conducted to optimize statistical parameters by combining different field contributions: steric (S) and electrostatic (E) fields for CoMFA, and steric (S), electrostatic (E), hydrophobic (H), hydrogen-bond donor (D), and hydrogen-bond acceptor (A) fields for CoMSIA (Supplementary Table S2).

As shown in Supplementary Table S2, the CoMFA models considered three field combinations, with CoMFA-SE exhibiting the best statistical performance, as indicated by the cross-validated correlation coefficient (q^2^) and standard error of prediction (SEP). In contrast, CoMSIA evaluated thirty-one field combinations, of which twenty-four had q^2^ values greater than 0.5, meeting previously established criteria (Golbraikh and Tropsha, 2002). The top-performing CoMSIA models based on q^2^ values were CoMSIA-SE (q^2^ = 0.808), CoMSIA-SEA (q^2^ = 0.789), and CoMSIA-SEH (q^2^ = 0.778), with optimal component numbers (N) of 5, 3, and 4, respectively. To rule out the possibility that the selected model was due to chance, a randomization test of the dependent variable (*Y*-random test) was performed. In this procedure, activity values in the dataset are randomly replaced. New models are then developed to check if similar or better statistical parameters can be obtained. In our case, after ten randomizations, the q^2^ values ranged from 0.095 to −0.534 (Supplementary Table S6). These are well below the acceptable threshold for a predictive 3D-QSAR model (q^2^ > 0.5). This confirms the robustness and statistical validity of the developed model.

As a secondary selection criterion, models with lower N values are preferred to avoid overfitting. It is generally accepted that the optimal number of components for 3D-QSAR models should not exceed one-tenth of the training set size (Tropsha, 2010; Roy et al., 2013); thus, four components were set as the maximum allowed for model selection. Consequently, the CoMSIA-SE model was excluded due to its high N value, and CoMSIA-SEH was rejected because of its lower q^2^ value. Therefore, the CoMSIA-SEA model (q^2^ = 0.789, N = 3) was selected as the most appropriate model for further analysis. The summary of the best CoMFA and CoMSIA models is reported in Supplementary Table S3. Both models demonstrated satisfactory predictive performance overall, with external r^2^ values exceeding 0.6 and CCC values above 0.70, indicating good agreement between predicted and experimental pIC_50_ values (Supplementary Table S5). The low MAE and RMSD values further support the reliability of the predictions for most compounds in both models. However, the presence of outliers in the test set—specifically compounds **017** and **018**—significantly affected the external validation metrics, resulting in a noticeable decrease in predictive statistics when these compounds were included in the calculations. These discrepancies could stem from unique electronic or steric features of the outlier molecules not fully captured by the models. Despite this, the overall performance of both CoMFA and CoMSIA remains robust and suitable for guiding further optimization of chalcone derivatives as potential antitumor agents.

**FIGURE 2:**
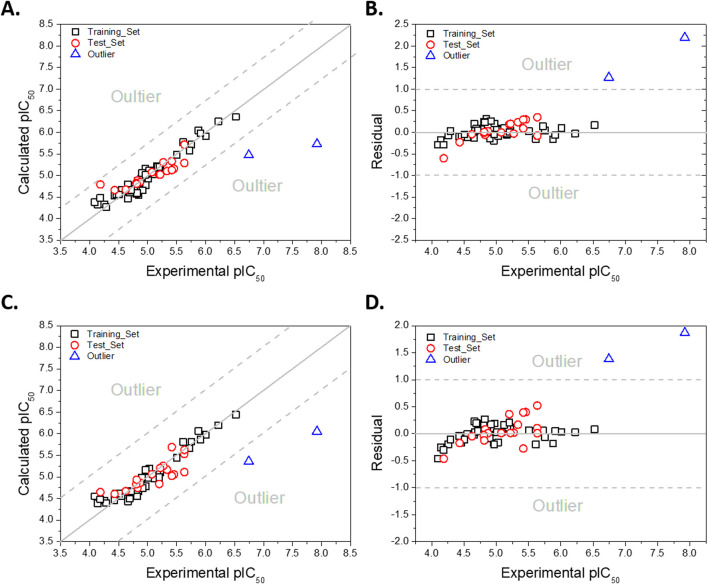
Plots of experimental versus predicted pIC_50_ values for the training (in black edge), test (in red edge) sets, and outlier compounds (in blue edge). **(A)** Experimental and predicted activity for CoMFA-SE model. **(B)** Variation of residual values for CoMFA-SE model. **(C)** Experimental and predicted activity for CoMSIA-SEA model. **(D)** Variation of residual values for CoMSIA-SEA model. Details are in Supplementary Table S4.

Following external validation, the experimental pIC_50_ values were plotted against the predicted values obtained from the CoMFA-SE and CoMSIA-SEA computational models (Figures 2A,C). Both plots demonstrate a strong correlation along the line *y* = *x* (shown in grey), except for two compounds identified as outliers (indicated by blue triangles). Additionally, Figures 2B,D present the residuals for each compound in the training and test sets for the corresponding models. The predicted values from both models yielded residuals below 1.0 logarithmic unit, indicating good predictive accuracy (Tropsha, 2010), except for the aforementioned outlier compounds.

**FIGURE 3:**
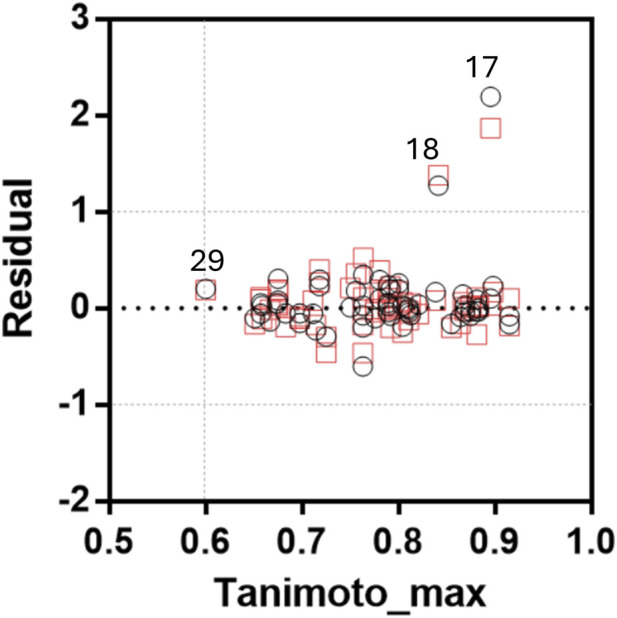
Plot of Tanimoto_max versus Residual (pIC_50 EXP_—pIC_50 PRED_) for CoMFA (black circles) and CoMSIA (red squares). Compounds **017** and **018** were biological outliers.

The applicability domain of the QSAR models was evaluated by plotting the maximum Tanimoto similarity index (Tanimoto_max) against the residuals for both CoMFA and CoMSIA models (Figure 3). A threshold of 0.60 was applied to Tanimoto_max to define the structural domain, while residuals exceeding ±1.0 log units were considered predictive outliers. Among the 64 compounds analyzed, only compound **029** was exactly at the structural similarity threshold (Tanimoto_max = 0.60), indicating that it lies at the boundary of the defined chemical space. However, no compound fell strictly outside the structural applicability domain. In terms of predictive performance, compounds **017** and **018** exhibited residuals exceeding ±1.0 log units in both CoMFA and CoMSIA models, indicating they are biological outliers. Interestingly, these two compounds exhibited Tanimoto_max values above 0.80, suggesting that they are well represented in the training set. A closer examination of their structures revealed significant differences in their electronic and physicochemical properties: compound **017** bears two strongly electron-withdrawing nitro groups, whereas compound **018** possesses a strongly electron-donating dimethylamino group on the phenyl ring. Such substituents can markedly alter the electronic distribution, conformational preferences, and potential receptor interactions. Moreover, nitro groups are well known to decrease compound solubility, which could affect bioavailability or experimental assay performance, potentially contributing to the observed discrepancies between experimental and predicted biological activities.

#### 3.1.1 Contour maps analysis

**FIGURE 4:**
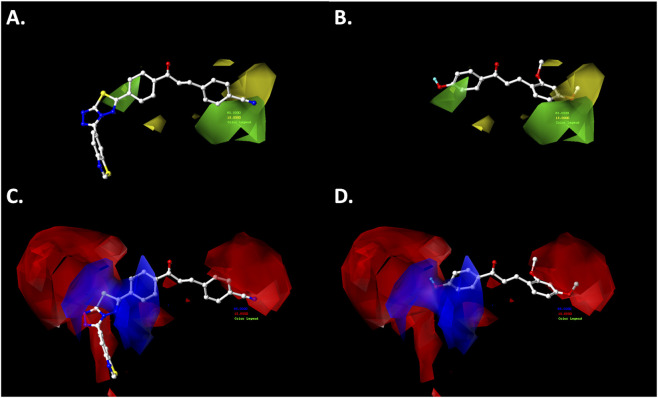
CoMFA steric **(A,B)** and electrostatic **(C,D)** contour maps around compounds **038** (left) and **021** (right), the most active and least active of the series, respectively. Color code: sterically favored (green) and disfavored (yellow). Electropositive favored (blue) and electronegative favored (red).

**FIGURE 5:**
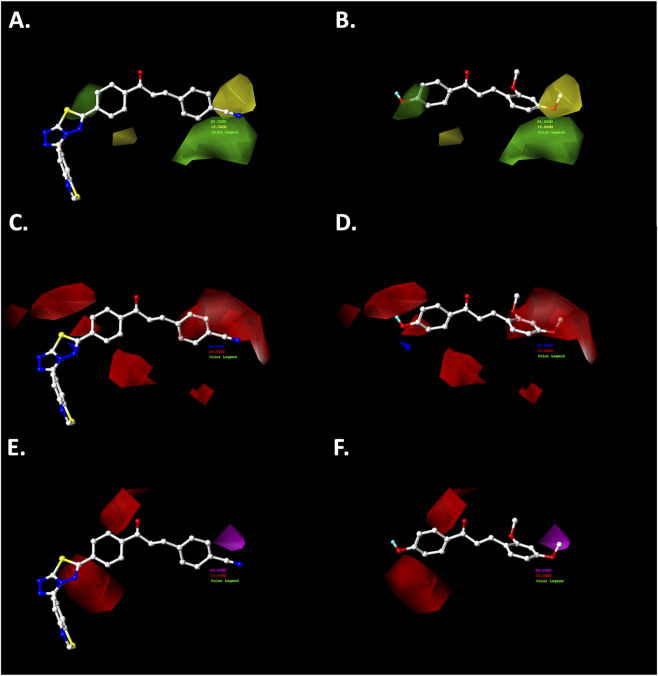
CoMSIA steric **(A,B)**, electrostatic **(C,D)**, and hydrogen-bonding acceptor **(E,F)** contour maps around compounds 038 (left) and 021 (right), the most and least active compounds in the series, respectively. Color code: sterically favored (green) and disfavored (yellow). Electropositive favored (blue) and electronegative favored (red). Hydrogen bonding acceptor favored (magenta), and disfavored (red).

The results of the CoMFA and CoMSIA models provide a set of steric, electrostatic, hydrophobic, and H-bonding features that can be strategically exploited for the rational design of next-generation chalcone derivatives. The contour maps and activity trends highlight key substitution patterns on the A and B rings that positively modulate antiproliferative activity. The contour maps generated from the theoretical CoMFA-SE and CoMSIA-SEA models highlight the most favorable substitution sites by color-coded polyhedra corresponding to different chemical properties. In this study, the cytotoxic effects of a dataset of 64 chalcone derivatives on ovarian cancer cells were investigated. The polyhedra were mapped around the most active compound in the training set, (*E*)-4-(3-(4-(3-(benzo[*d*]thiazol-6-yl)-[1,2,4]triazolo[3,4-*b*][1,3,4]thiadiazol-6-yl)phenyl)-3-oxoprop-1-en-1-yl)benzonitrile (compound **038**, pIC_50_ = 6.523), and the least active compound, (*E*)-3-(2,4-dimethoxyphenyl)-1-(4-hydroxyphenyl)prop-2-en-1-one (compound **021**, pIC_50_ = 4.089 M). The corresponding results are shown in Figures 4, 5.

#### 3.1.2 Steric map

The steric contour maps generated from both the CoMFA and CoMSIA models exhibited a high degree of similarity (Figures 4A,B, 5A,B). In the A-ring of the chalcone core, a green polyhedron is observed near the *meta-* and *para-*positions, indicating that bulky substituents at these sites enhance the growth-inhibitory activity against the A2780 cancer cell line. Notably, the most active compound (**038**) features a heterocyclic substituent in the *para* position—3-(benzo[*d*]thiazol-6-yl)-[1,2,4]triazolo[3,4-*b*][1,3,4]thiadiazol-6-yl—bonded via a single bond, which allows free rotation to optimally orient this fragment within the favorable steric region, thereby enhancing antiproliferative activity. In contrast, the less active compound (**021**) possesses a hydroxyl group at the *para*-position of the A-ring, which, despite being located within the favorable green polyhedron, lacks sufficient bulk to improve cytotoxic effects significantly.

Regarding the B-ring, a yellow polyhedron is projected near the *para*-position, suggesting that bulky substituents at this site reduce growth-inhibitory activity against the A2780 cell line. This observation is consistent with the less active compound (**021**), which bears a methoxy (−OCH_3_) substituent in this region. The single bond and angular geometry of the methoxy group (C_Ar_–O–CH_3_) permit free rotation, increasing steric bulk compared to the linear nitrile (−C≡N) substituent present in the most active compound (**038**). This latter substituent is positioned near a green polyhedron, consistent with its favorable contribution to biological activity.

#### 3.1.3 Electrostatic map

The electrostatic maps derived from the CoMFA and CoMSIA analyses exhibited a high degree of similarity, particularly in the B-ring of the chalcone pharmacophoric core (Figures 4C,D, 5C,D). Near the *meta-* and *para-*positions, red polyhedra are projected, indicating that electron-rich substituents at these positions enhance the antiproliferative effects against the A2780 cancer cell line. For example, the most active compound in the dataset (**038**) positions the nitrogen atom of the nitrile group (−CN) in this favorable region. In contrast, the less active compound places a carbon atom from the methoxy group (-OCH_3_) in this area, which is relatively electron-deficient due to the oxygen atom’s electronegativity.

Conversely, the electrostatic map of the A-ring shows a red polyhedron at the *meta*-position, suggesting that electron-rich substituents at this position favor antiproliferative activity against ovarian cancer cells. In the most active compound (**038**), the substituent 3-(benzo[*d*]thiazol-6-yl)-[1,2,4]triazolo[3,4-*b*][1,3,4]thiadiazol-6-yl is bonded at the *para*-position via a single bond, allowing rotational flexibility that orients the heterocyclic fragment toward this electron-rich region. Conversely, the less active compound (**021**) features a hydroxyl group at the *para*-position, which aligns with a blue polyhedron, indicating that electron-rich substituents in this position reduce antiproliferative activity against the A2780 ovarian cancer cell line.

#### 3.1.4 Hydrogen bonding acceptor map

The hydrogen bond acceptor (HBA) map derived from the CoMSIA model (Figures 5E,F) reveals a red polyhedron near the *ortho-* and *para*-positions of the A-ring, indicating that the presence of an HBA substituent in these positions does not enhance cytotoxic activity against the A2780 cancer cell line. For instance, in the most active compound (**038**), the heterocyclic fragment [1,2,4]triazolo[3,4-*b*][1,3,4]thiadiazol-6-yl is oriented near the *para*-position, positioning sulfur and nitrogen atoms close to this red polyhedron, which correlates with increased biological activity (Figure 5E).

In contrast, the B-ring exhibits a magenta polyhedron near the *meta*-position, suggesting that an HBA substituent at this site favors antiproliferative activity against the A2780 cell line. Although the most active compound (**038**) lacks a substituent at the *meta*-position, the less active compound (**021**) positions the methyl group of its methoxy substituent (-OCH_3_) within this magenta polyhedron, consistent with its relatively low cytotoxic effect.

#### 3.1.5 Summary of the principal results from the computational models and design

**FIGURE 6:**
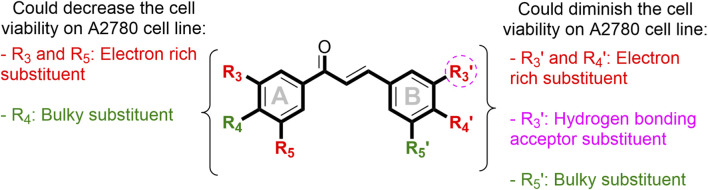
Summary of the main structure–activity relationships found in this study using 3D-QSAR analysis.

From the 3D-QSAR models developed using CoMFA and CoMSIA, based on steric, electrostatic, and hydrogen-bonding acceptor fields. These most critical structural features that enable antiproliferative activity in the ovarian cancer cell line A2780 have been identified. This information is summarized in Figure 6.

### 3.2 Experimental validation of 3D-QSAR models

**FIGURE 7:**
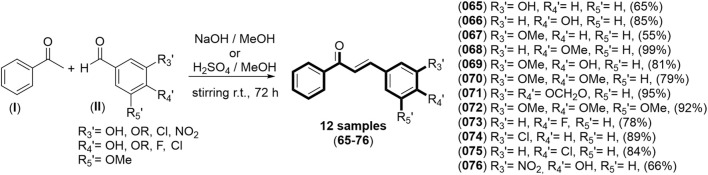
Synthetic route to obtain the experimental test set of 3D-QSAR models. The yield of each product is displayed in parentheses. **I** Acetophenone; **II** Substituted benzaldehyde.

Regarding the summary of the key chemical features of substituents attached to the chalcone core that modulate antiproliferative activity against the A2780 ovarian cancer cell line (Figure 6), the results for the B-ring were consistently supported by both CoMFA and CoMSIA analyses (Figures 4, 5). Consequently, the design of chalcone derivatives focused on structural modifications of the B-ring while maintaining an unsubstituted A-ring. Based on this criterion, electron-rich substituents such as –OH, –OR, and halogens, as well as hydrogen bond acceptor groups (*e.g.,* –NO_2_), were proposed for the R_3_’ position on the B-ring. Similarly, electron-rich substituents (*e.g.,* –OH, –OR, and halogens) were suggested for the R_4_’ position. Furthermore, due to the single bond between the β-conjugated carbon and the B-ring, substitution at position R_3_’ can be alternatively introduced at position R_5_’. Therefore, bulky and electron-rich substituents were proposed for the R_5_’ position. The structures of the proposed chalcone derivatives are illustrated in Figure 7.

For the synthesis of the proposed chalcone derivatives, acetophenone (**I**) was reacted with the appropriate benzaldehyde (**II**) under either alkaline or acidic conditions, catalyzed by the Claisen-Schmidt reaction, to obtain the desired compounds (Bhagat et al., 2006; Kumar and Akanksha, 2007; Narender and Papi Reddy, 2007). The identities of the synthesized compounds were confirmed using classical spectroscopic techniques, including Fourier transform infrared (FT-IR) and nuclear magnetic resonance (NMR) spectroscopy. In the FT-IR spectra, all compounds exhibited a characteristic absorption band at approximately ῡ = 1,615 cm^-1^, attributable to the stretching vibration of the conjugated carbonyl group. Additionally, depending on the substituents attached at positions R_3_’, R_4_’, and R_5_’, the FT-IR spectra displayed signals corresponding to hydroxyl groups (Ph–OH, ῡ ∼3,300 cm^-1^), methoxyl and dioxomethylene groups (–OCH_3_, –OCH_2_O–, ῡ = 1,250 cm^-1^), fluorine (–F, ῡ = 1,450 cm^-1^), chlorine (–Cl, ῡ = 750 cm^-1^), and nitro groups (–NO_2_, ῡ = 1,550 and ῡ = 1,530 cm^-1^) (Organic Molecules, 2004).

In the ^1^H-NMR spectra, each synthesized compound showed two downfield doublets at approximately δ = 7.76 ppm and δ = 7.52 ppm, each with a coupling constant of around *J* = 15.4 Hz. These signals correspond to the vinyl hydrogens in the *trans* configuration adjacent to the carbonyl group and the B-ring, which are characteristic of chalcone structures (Mellado et al., 2020).

Following synthesis and characterization, the designed compounds, guided by 3D-QSAR models, were evaluated for cytotoxicity against the A2780 ovarian cancer cell line. The experimental results were compared with the theoretical predictions obtained from the CoMFA-SE and CoMSIA-SEA models. A summary of these results is presented in Table 1.

**TABLE 1:** Experimental and predicted activity by computational models for compounds **065–076** against ovarian cancer cell line A2780.

Compound	Experimental values		Calculated values			
	IC_50_ (µM)	pIC_50_	CoMFA-SE	Residual	CoMSIA-SEA	Residual
**065**	19.69 ± 2.75*	4.706	4.710	−0.004	4.781	−0.075
**066**	24.95 ± 5.15*	4.603	4.625	−0.022	4.730	−0.127
**067**	40.16 ± 4.69*	4.396	4.455	−0.059	4.605	−0.209
**068**	185.20 ± 19.46	3.732	4.429	−0.697	4.399	−0.667
**069**	127.6 ± 6.15	3.894	4.575	−0.681	4.736	−0.842
**070**	60.79 ± 2.22	4.216	4.621	−0.405	4.537	−0.321
**071**	49.37 ± 2.30*	4.307	4.573	−0.266	4.737	−0.430
**072**	23.12 ± 2.66*	4.636	4.631	0.005	4.688	−0.052
**073**	86.56 ± 5.22	4.063	4.689	−0.626	4.723	−0.660
**074**	37.97 ± 2.62*	4.421	4.665	−0.244	4.713	−0.292
**075**	59.02 ± 9.69	4.229	4.571	−0.342	4.754	−0.525
**076**	179.9 ± 21.88	3.745	4.558	−0.813	4.603	−0.858
Cisplatin	22.56 ± 1.19*	-	-	-	-	-

*p > 0.05.

Table 1 shows that the predicted activity values from both models (CoMFA-SE and CoMSIA-SEA) are slightly overestimated, as indicated by the negative residual values. Despite this, the residuals for all assessed compounds remain below 1.0 logarithmic unit, demonstrating good agreement with the theoretical models. Notably, the highest antiproliferative activities against the A2780 cancer cell line were observed for (*E*)-3-(3-hydroxyphenyl)-1-phenylprop-2-en-1-one (**065**, pIC_50_ = 4.706) and (*E*)-1-phenyl-3-(3,4,5-trimethoxyphenyl)prop-2-en-1-one (**072**, pIC_50_ = 4.636).

The antiproliferative activity observed for compound **065** (pIC_50_ = 4.706) aligns well with its close structural resemblance to compounds **046** (pIC_50_ = 5.418), **047** (pIC_50_ = 5.122), **049** (pIC_50_ = 5.199), and **054** (pIC_50_ = 5.218). These analogues possess bulky, electron-rich substituents at the R_3_′ position of ring B (e.g., –OH, –F, and –Br), a feature that appears to enhance antiproliferative potency within this chemical series. Consistent with this structural pattern, these compounds exhibit pIC_50_ values ranging from 5.122 to 5.418 (Supplementary Table S4), in agreement with trends previously described for chalcone derivatives (Pan et al., 2024).

Compound **072** (pIC_50_ = 4.636) contains a 3′,4′,5′-trimethoxy substitution pattern on ring B, which mirrors that of compound **030** (pIC_50_ = 5.456) and is closely related to the 3′,5′-dimethoxy arrangement found in compound **034** (pIC_50_ = 6.222). The recurrence of these methoxy substitution patterns among active analogues is consistent with the overall structure–activity trend, in which electron-donating groups on ring B contribute favorably to antiproliferative potency (Qi et al., 2014; Bujji et al., 2020). This alignment reinforces the predictive capacity of the QSAR-guided design strategy.

In contrast, the less active compounds, **068** and **076**, display substitution patterns on ring B that are less favorable for antiproliferative activity (4′-OMe and 3′-NO_2_/4′-OH, respectively). Compound **068** (pIC_50_ = 3.732) shows partial structural consistency with compounds **041**–**044** and **053**, which incorporate electron-rich substituents of different sizes (–F, –Cl, –Br, –I, –OH) and exhibit activity within the pIC_50_ range of 4.622–5.090 (Supplementary Table S4) (Pan et al., 2024). Meanwhile, compound **076** (pIC_50_ = 3.745), bearing a nitro group at the 3′ position and a hydroxyl group at 4′, resembles other low-activity analogues, such as compounds **028**–**029** (3′-CF_3_; pIC_50_ = 4.847 and 4.973) and compound **053** (4′-OH; pIC_50_ = 4.622). This concordance suggests that specific electron-rich substitutions at these positions tend to reduce antiproliferative potency within this chalcone series.

**FIGURE 8:**
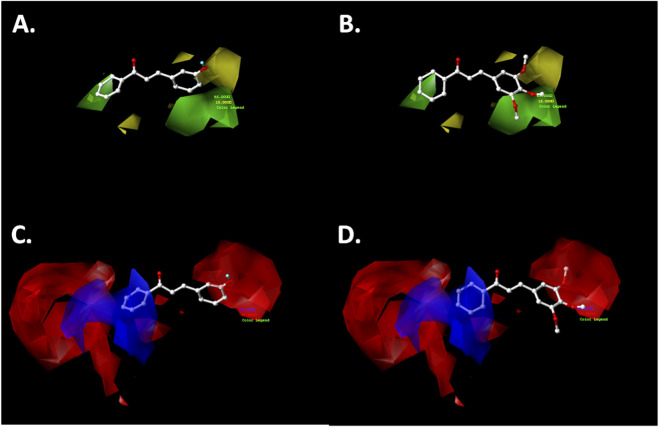
CoMFA steric **(A,B)** and electrostatic **(C,D)** contour maps around the compounds **065** (left) and **072** (right), the most active in the experimental series. Color code: sterically favored (green) and disfavored (yellow). Electropositive favored (blue) and electronegative favored (red).

**FIGURE 9:**
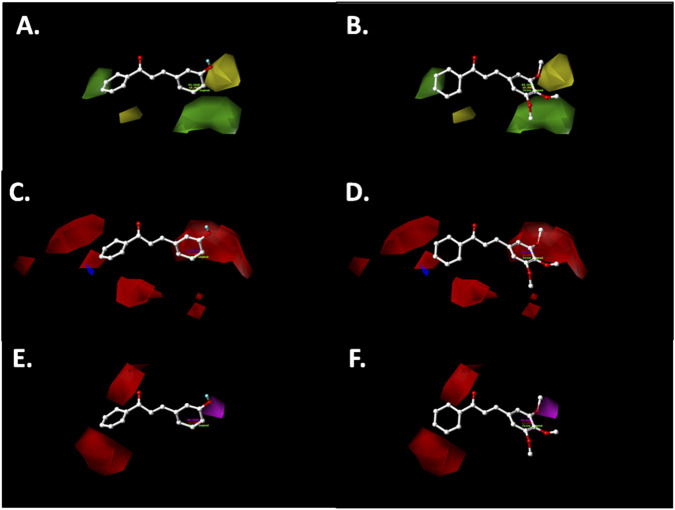
CoMSIA steric **(A,B)**, electrostatic **(C,D)**, and hydrogen bonding acceptor **(E,F)** contour maps around compounds **065** (left) and **072** (right), the most active in the experimental series. Color code: sterically favored (green) and disfavored (yellow). Electropositive favored (blue) and electronegative favored (red). Hydrogen bonding acceptor favored (magenta), and disfavored (red).

The most active compounds were further analyzed using contour maps, as shown in Figures 8, 9.

The steric map of (*E*)-3-(3-hydroxyphenyl)-1-phenylprop-2-en-1-one (**065**) shows the hydroxyl group oriented away from the yellow polyhedron (Figure 8A). In contrast, the methoxy groups (–OCH_3_) at the *meta-* and *para*-positions of the B-ring in (*E*)-1-phenyl-3-(3,4,5-trimethoxyphenyl)prop-2-en-1-one (**072**) are directed toward the green polyhedron, which favors the inhibitory activity against the A2780 cancer cell line (Figure 8B).

Regarding the electrostatic maps from both CoMFA and CoMSIA models (Figures 8, 9), the hydroxyl (–OH) and methoxy (–OCH_3_) groups at the *meta*-position of the B-ring are projected onto the red polyhedron, indicating that the presence of these electron-rich substituents enhances cytotoxic activity against the ovarian cancer cell line.

Furthermore, the hydrogen bonding acceptor (HBA) map from the CoMSIA model (Figures 9E,F) shows that the hydroxyl group (–OH) in compound **065** is directed toward the magenta polyhedron, favoring antiproliferative activity. Similarly, in compound **072**, the *meta*-methoxy group (–OCH_3_) exhibits a comparable effect.

Experimental validation of selected derivatives confirms the models’ predictive value and supports their use to guide future chemical modifications aimed at improving activity against drug-sensitive ovarian cancer cells.

### 3.3 Evaluation of the potential cell death mechanism

**FIGURE 10:**
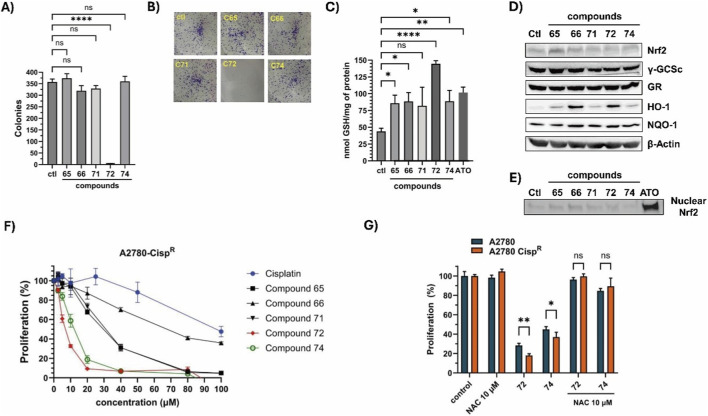
Antiproliferative effects and redox imbalance caused by selected chalcone derivatives in human ovarian cancer cells. **(A)** A2780 cells were treated with the indicated compounds (20 μM) for 24 h. The clonogenic potential was then assessed as described in the Materials and Methods section. Bars show the number of colonies formed under each treatment condition (means ± SD, *n* = 3; ****p ≤ 0.0001; ns: not significant). **(B)** Representative images of the clonogenic assay conducted on standard 6-well plates are provided (original images in Supplementary Figures S3–S8). **(C)** Intracellular GSH levels (nmol GSH/mg protein) were measured in A2780 cells treated with the indicated compounds (20 μM) or ATO (5 μM) for 24 h. Statistically significant differences are marked (mean ± SD, *n* = 3; **p ≤ 0.01; ****p ≤ 0.0001). **(D)** A2780 cells were treated with the indicated compounds (20 μM) for 24 h. Total protein extracts were used to evaluate the expression levels of Nrf2, γ-GCSc, GR, HO-1, NQO-1, and β-Actin by Western blot. Representative blots are shown (original images in Supplementary Figures S9–S15). **(E)** Nrf2 was detected in nuclear extracts of A2780 cells treated with the selected compounds (20 µM) for 24 h. A representative blot is shown. **(F)** A2780-Cisp^R^ cells were treated with different concentrations (2.5 μM–100 μM) of selected chalcone derivatives (**065**, **066**, **071**, **072**, or **074**) or cisplatin for 24 h. Cell proliferation was measured using the MTT assay. The curves illustrate the percentage of proliferation relative to the control condition (0.1% DMSO), calculated from absorbance measurements at 570 nm in at least three independent experiments (means ± SD). **(G)** A2780 and A2780 Cisp^R^ cells were treated with compounds **072** or **074** in the presence or absence of NAC (10 μM) for 24 h. Proliferation was assessed by the MTT assay as described (means ± SD, *n* = 3).

As a supplement to the antiproliferative effects of chalcone derivatives observed in A2780 cells, we investigated how the most potent compounds—those with the lowest IC_50_ values in the screening panel—affect the ability of individual cancer cells to grow and form visible colonies (clonogenic assay). As shown in Figures 10A,B, the clonogenic capacity of A2780 cells was suppressed by 24-h treatment with compound **072**, whereas the other compounds showed no significant difference relative to control (Supplementary Figures S3–S8). This unexpected result prompted us to investigate whether these compounds could affect the cellular redox state, with a focus on GSH, a key redox molecule. As shown in Figure 10C, all compounds significantly increased GSH levels in A2780 cells, with compound **072** causing a 3.2-fold increase over basal levels. A similar effect was seen with arsenic trioxide (ATO, 5 µM), a well-known prooxidant agent (Yan et al., 2024). Based on this, we hypothesized that the increase in GSH might be due to enhanced Nrf2 activity, a master regulator of the antioxidant response to stress (Zhang, 2025). However, as shown in Figure 10D, Nrf2 basal protein levels remained unchanged following treatment with any of the compounds compared to the control condition (Supplementary Figure S14). Further analysis of Nrf2 nuclear accumulation by Western blot revealed that none of the compounds promoted its translocation, except for arsenic trioxide, as expected (Figure 10E; Supplementary Figure S15). Conversely, the enzyme HO-1, typically induced by oxidative stress and considered a hallmark of arsenic exposure (Valenzuela et al., 2014), was upregulated by all compounds, with the most potent effects observed with compounds **066** and **072** (Figure 10D; Supplementary Figure S12). In contrast, other canonical Nrf2 transcriptional targets, including γ-GCSc, NQO1, and GR, were not induced by any of the tested compounds (Supplementary Figures S10, S11, S13, respectively).

We next asked whether this series of compounds was effective against a cisplatin-resistant ovarian cancer cell line. To this end, we generated a resistant cell line by chronic exposure to increasing concentrations of cisplatin. The A2780-Cisp^R^ line was then treated with various doses of the compounds and cisplatin. As shown in Figure 10F, the MTT assay revealed that this cell line was more sensitive to compounds **072** and **074** than the parental line, with IC_50_ values for compounds **065**, **066**, **071**, **072**, and **074** of 27.79 ± 2.59 µM, 74.41 ± 5.68 µM, 29.50 ± 2.74 µM, 6.50 ± 0.34 µM, and 10.22 ± 1.12 µM, respectively. In contrast, cisplatin showed an IC_50_ of 93.4 ± 1.40 µM in this line—approximately four times higher than in the parental line A2780—confirming its resistant phenotype.

Finally, we evaluated the involvement of ROS in the cytotoxicity induced by compounds **072** and **074**. As shown in Figure 10G, the antioxidant molecule *N*-acetylcysteine (NAC) completely abrogated the cytotoxic effect of compounds **072** and **074**, indicating that the two compounds may act through ROS-mediated mechanisms. Taken together, these data demonstrate the anti-tumor potential of chalcone-derived compounds in ovarian cancer, with compounds **072** and **074** showing auspicious activity.

The differences observed between parental A2780 and cisplatin-resistant A2780-CispR cells are consistent with resistance-associated adaptive mechanisms that are widely documented in ovarian cancer. Cisplatin-resistant models often exhibit enhanced glutathione biosynthesis, increased Nrf2-mediated antioxidant responses, and upregulation of detoxification enzymes and efflux transporters, all of which attenuate the cytotoxic effects of electrophilic or ROS-inducing compounds (Godwin et al., 1992; Galluzzi et al., 2012; Traverso et al., 2013; Rojo de la Vega et al., 2018). These adaptations provide a plausible explanation for the reduced potency of compounds such as **065** and **071** in the CispR context, whereas derivatives like **072** and **074** appear less susceptible to intracellular antioxidant buffering.

While our study primarily focuses on the development and robust validation of 3D-QSAR models (CoMFA and CoMSIA) to predict antiproliferative activity against the A2780 cell line (representative of an endometrioid phenotype), we recognize that their applicability may be limited when extrapolated to other ovarian cancer subtypes. High-grade serous ovarian cancer (HGSOC), which accounts for approximately 70% of cases, exhibits substantial genomic instability and distinct chemoresistance mechanisms, features typically modeled in lines such as SKOV-3 or OVCAR-3 (Domcke et al., 2013; Beaufort et al., 2014). Extending the QSAR models to these subtypes, potentially by incorporating more diverse datasets, would broaden their predictive scope and better capture subtype-specific biological determinants.

Despite these constraints, the strong statistical performance of our models (q^2^ > 0.76; r^2^ > 0.92), together with the successful experimental validation of 12 synthesized chalcones, supports their reliability for guiding rational design. The contour maps generated by CoMFA and CoMSIA highlight key structural features such as bulky, electron-rich substituents at the *meta* and *para* positions of the B ring, that consistently enhance antiproliferative activity. These insights provide a clear blueprint for future optimization of chalcone scaffolds or for the development of hybrid analogues, similar to quinoline–chalcone hybrids previously reported to inhibit tubulin polymerization in ovarian cancer cells (Mirzaei et al., 2020).

Our mechanistic observations further support the relevance of redox-related pathways in mediating the activity of these compounds. The partial reversal of cytotoxicity by NAC and the upregulation of HO-1 in the absence of canonical Nrf2 activation are consistent with reports that chalcone-induced oxidative stress drives antiproliferative effects in cancer cells resistant to canonical Nrf2 activation (Qi et al., 2014). Taken together, these findings underscore that, although currently limited to the A2780 model, our 3D-QSAR framework provides a strong foundation for iterative structure-guided optimization and may accelerate the discovery of chalcone-based therapeutics across diverse ovarian cancer phenotypes.

### 3.4 Challenges and limitations

The present findings are consistent with previous evidence positioning chalcones as multitarget antiproliferative agents; however, this work extends the current understanding by linking specific steric and electrostatic determinants to redox modulation in ovarian cancer cells. The enhanced activity observed for derivatives bearing bulky electron-withdrawing substituents at the para-position of ring B corroborates prior 3D-QSAR analyses conducted in other tumor models, thereby reinforcing the predictive robustness of the CoMFA and CoMSIA models developed herein (ElMchichi et al., 2020; Rudrapal et al., 2021). Notably, compounds **072** and **074** exhibited more potent cytotoxic effects in cisplatin-resistant A2780 cells than cisplatin itself, in alignment with prior reports describing chalcones capable of overcoming platinum resistance through oxidative stress induction and inhibition of STAT3 or NF-κB signaling pathways (Qi et al., 2014; Gozari et al., 2021). Moreover, the selective upregulation of HO-1 in the absence of canonical Nrf2 nuclear translocation suggests a non-classical adaptive response. This contrasts with studies in which Nrf2 hyperactivation promoted cytoprotection (Leon-Gonzalez et al., 2015; Michalkova et al., 2023) and may reflect differences in electrophilic strength or context-dependent modulation of thiol-based sensors, a phenomenon previously described for Michael acceptors (Kenari et al., 2021).

Despite these strengths, several limitations should be explicitly acknowledged. First, the chemical space investigated is restricted to chalcone-like scaffolds and closely related derivatives. This limited structural diversity inherently constrains the applicability domain of the CoMFA/CoMSIA models and reduces the reliability of predictions when extrapolated to chemically distant scaffolds (Gramatica, 2007; Tropsha, 2010). Second, 3D-QSAR approaches are intrinsically dependent on molecular alignment and conformational choices. Variability introduced during the alignment process, selection of reference conformations, and the handling of flexible substituents may influence contour map generation and, consequently, the interpretation of steric and electrostatic hotspots (Urniaż and Jóźwiak, 2013; Wilkes et al., 2016). Third, although CoMFA and CoMSIA contour maps provide valuable visual guidance for scaffold optimization, their interpretation can be challenging and may involve subjectivity, particularly when evaluating overlapping or spatially diffuse polyhedra (Lorca et al., 2024).

Furthermore, the 3D-QSAR models exhibited strong internal (cross-validation) and external (test set) predictive performance for the A2780 cell line. However, their applicability is restricted to this specific biological context. Structure–activity relationships governing chalcone sensitivity can differ markedly across ovarian cancer subtypes (even within the same organ), thus precluding direct extrapolation to other cellular systems without independent datasets and validation. This limitation is inherent to QSAR methodologies, which rely on phenotype-dependent descriptors that may not be conserved across heterogeneous tumor landscapes.

Finally, the experimental arm of the study is limited by the modest number of compounds evaluated (*n* = 12) and by the absence of pharmacokinetic, metabolic, or *in vivo* assessments. These factors constrain the ability to establish translational relevance or predict therapeutic indices in physiological settings. Nonetheless, the integration of computational modeling with mechanistic assays provides a rigorous framework for the rational design of chalcone-based derivatives that combine redox-mediated cytotoxicity with activity in chemoresistant ovarian cancer. This combined approach lays a strong foundation for iterative optimization cycles and may accelerate the discovery of clinically relevant chalcone-derived therapeutics across diverse ovarian cancer phenotypes.

## 4 Conclusion

In this work, CoMFA and CoMSIA 3D-QSAR models were successfully developed for a series of 64 chalcone derivatives evaluated against the A2780 ovarian cancer cell line. The CoMFA model achieved a cross-validated correlation coefficient (q^2^) of 0.763 and an r^2^ of 0.963, while the CoMSIA model exhibited a q^2^ of 0.789 and an r^2^ of 0.920, demonstrating robust predictive performance. Steric and electrostatic contour maps revealed that bulky substituents and electron-withdrawing groups at the *para*-position of the aromatic rings significantly enhance antiproliferative activity. Moreover, the experimental synthesis and biological testing of 12 new chalcones yielded compounds with predicted pIC_50_ values that closely matched the experimental results, validating the models’ applicability. Based on insights from CoMFA and CoMSIA models, rationally designed chalcones were proposed as promising anticancer agents against ovarian cancer cells. Among the newly synthesized compounds, molecules **065**, **066**, and **072** exhibited the highest pIC_50_ values. Furthermore, compounds **072** and **074** showed effective antiproliferative activity against cisplatin-resistant ovarian cancer cells.

Additionally, compound **072** significantly suppressed clonogenic growth in A2780 cells, further confirming its potent antiproliferative capacity. Mechanistically, the most active derivatives elevated intracellular GSH levels and consistently upregulated HO-1 expression without activating the canonical Nrf2 pathway, suggesting a selective redox-modulating activity. Importantly, their cytotoxicity was abrogated by NAC, indicating a ROS-dependent mechanism of action. These findings highlight not only the predictive power of the 3D-QSAR models but also the therapeutic promise of chalcone derivatives as leads against both sensitive and drug-resistant ovarian cancer phenotypes.

The integrated computational and experimental approach presented here provides a valuable platform for guiding the design of potent, mechanistically insightful chalcone derivatives targeting ovarian cancer, with some compounds showing cytotoxicity comparable to that of cisplatin and maintaining activity in cisplatin-resistant ovarian cancer cells.

## Data Availability

The original contributions presented in the study are included in the article/Supplementary Material, further inquiries can be directed to the corresponding authors.

## References

[B1] Adhikari S. Nath P. Deb V. K. Das N. Banerjee A. Pathak S. (2025). Pharmacological potential of natural chalcones: a recent studies and future perspective. Front. Pharmacol. 16, 1570385. 10.3389/fphar.2025.1570385 40599794 PMC12209288

[B2] Bajusz D. Rácz A. Héberger K. (2015). Why is tanimoto index an appropriate choice for fingerprint-based similarity calculations? J. Cheminform. 7, 20. 10.1186/s13321-015-0069-3 26052348 PMC4456712

[B3] Beaufort C. M. Helmijr J. C. A. Piskorz A. M. Hoogstraat M. Ruigrok-Ritstier K. Besselink N. (2014). Ovarian cancer cell line panel (OCCP): clinical importance of *in vitro* morphological subtypes. PLoS One 9, e103988. 10.1371/journal.pone.0103988 25230021 PMC4167545

[B4] Berridge M. V. Herst P. M. Tan A. S. (2005). “Tetrazolium dyes as tools in cell biology: new insights into their cellular reduction,” in Biotechnology annual review, 127–152. 10.1016/S1387-2656(05)11004-7 16216776

[B5] Bhagat S. Sharma R. Sawant D. M. Sharma L. Chakraborti A. K. (2006). LiOH·H2O as a novel dual activation catalyst for highly efficient and easy synthesis of 1,3-diaryl-2-propenones by claisen–schmidt condensation under mild conditions. J. Mol. Catal. A Chem. 244, 20–24. 10.1016/j.molcata.2005.08.039

[B6] Bujji S. Edigi P. K. Subhashini N. J. P. (2020). Synthesis and evaluation of novel 1,2,4‐triazolo‐[3,4‐b]‐1,3,4‐thiadiazole tethered chalcone hybrids as potential anticancer agents. J. Heterocycl. Chem. 57, 3318–3325. 10.1002/jhet.4047

[B7] Bustos L. Echiburú-Chau C. Castro-Alvarez A. Bradshaw B. Simirgiotis M. J. Mellado M. (2022). Cytotoxic effects on breast cancer cell lines of chalcones derived from a natural precursor and their molecular docking analysis. Molecules 27, 4387. 10.3390/molecules27144387 35889260 PMC9318862

[B8] Carrasco N. Garrido M. Montenegro I. Madrid A. Hartley R. González I. (2023). Antitumoral activity of Leptocarpha rivularis flower extracts against gastric cancer cells. Int. J. Mol. Sci. 24, 1439. 10.3390/ijms24021439 36674960 PMC9862749

[B9] Clark M. Cramer R. D. Van Opdenbosch N. (1989). Validation of the general purpose tripos 5.2 force field. J. Comput. Chem. 10, 982–1012. 10.1002/jcc.540100804

[B10] Cramer R. D. Patterson D. E. Bunce J. D. (1988). Comparative molecular field analysis (CoMFA). 1. Effect of shape on binding of steroids to carrier proteins. J. Am. Chem. Soc. 110, 5959–5967. 10.1021/ja00226a005 22148765

[B11] Domcke S. Sinha R. Levine D. A. Sander C. Schultz N. (2013). Evaluating cell lines as tumour models by comparison of genomic profiles. Nat. Commun. 4, 2126. 10.1038/ncomms3126 23839242 PMC3715866

[B12] Ducki S. Rennison D. Woo M. Kendall A. Chabert J. F. D. McGown A. T. (2009). Combretastatin-like chalcones as inhibitors of microtubule polymerization. Part 1: synthesis and biological evaluation of antivascular activity. Bioorg. Med. Chem. 17, 7698–7710. 10.1016/j.bmc.2009.09.039 19837593

[B13] Elkanzi N. A. A. Hrichi H. Alolayan R. A. Derafa W. Zahou F. M. Bakr R. B. (2022). Synthesis of chalcones derivatives and their biological activities: a review. ACS Omega 7, 27769–27786. 10.1021/acsomega.2c01779 35990442 PMC9386807

[B14] Elkhalifa D. Siddique A. B. Qusa M. Cyprian F. S. El Sayed K. Alali F. (2020). Design, synthesis, and validation of novel nitrogen-based chalcone analogs against triple negative breast cancer. Eur. J. Med. Chem. 187, 111954. 10.1016/j.ejmech.2019.111954 31838326

[B15] ElMchichi L. Belhassan A. Lakhlifi T. Bouachrine M. (2020). 3D-QSAR study of the chalcone derivatives as anticancer agents. J. Chem. 2020, 1–12. 10.1155/2020/5268985

[B16] Galluzzi L. Senovilla L. Vitale I. Michels J. Martins I. Kepp O. (2012). Molecular mechanisms of cisplatin resistance. Oncogene 31, 1869–1883. 10.1038/onc.2011.384 21892204

[B17] Godwin A. K. Meister A. O’Dwyer P. J. Huang C. S. Hamilton T. C. Anderson M. E. (1992). High resistance to cisplatin in human ovarian cancer cell lines is associated with marked increase of glutathione synthesis. Proc. Natl. Acad. Sci. 89, 3070–3074. 10.1073/pnas.89.7.3070 1348364 PMC48805

[B18] Golbraikh A. Tropsha A. (2002). Beware of q2. J. Mol. Graph. Model. 20, 269–276. 10.1016/S1093-3263(01)00123-1 11858635

[B19] González-Vergara A. Sánchez-González R. Bravo M. A. Aguilar L. F. Espinoza L. Mellado M. (2022). Assessment of chalcone-vanillin as a selective chemosensor of As(III) in aqueous solution. J. Mol. Struct. 1266, 133558. 10.1016/j.molstruc.2022.133558

[B20] Gozari M. Alborz M. El-Seedi H. R. Jassbi A. R. (2021). Chemistry, biosynthesis and biological activity of terpenoids and meroterpenoids in bacteria and fungi isolated from different marine habitats. Eur. J. Med. Chem. 210, 112957. 10.1016/j.ejmech.2020.112957 33160760

[B21] Gramatica P. (2007). Principles of QSAR models validation: internal and external. QSAR Comb. Sci. 26, 694–701. 10.1002/qsar.200610151

[B22] Janse van Rensburg H. D. Legoabe L. J. Terre’Blanche G. (2021). C3 amino-substituted chalcone derivative with selective adenosine rA1 receptor affinity in the micromolar range. Chem. Pap. 75, 1581–1605. 10.1007/s11696-020-01414-9 PMC7670844 33223599

[B23] Jeon J.-H. Kim S.-J. Kim C.-G. Kim J.-K. Jun J.-G. (2012). Synthesis of biologically active chalcones and their anti-inflammatory effects. Bull. Korean Chem. Soc. 33, 953–957. 10.5012/bkcs.2012.33.3.953

[B24] Jiang Q. Jia J. Xu B. Zhao A. Guo C.-C. (2015). Iron-facilitated oxidative radical decarboxylative cross-coupling between α-Oxocarboxylic acids and acrylic acids: an approach to α,β-Unsaturated carbonyls. J. Org. Chem. 80, 3586–3596. 10.1021/acs.joc.5b00267 25757053

[B25] Jin J. Qiu S. Wang P. Liang X. Huang F. Wu H. (2019). Cardamonin inhibits breast cancer growth by repressing HIF-1α-dependent metabolic reprogramming. J. Exp. Clin. Cancer Res. 38, 377. 10.1186/s13046-019-1351-4 31455352 PMC6712736

[B26] Jin Y.-H. Min J. S. Kwon S. (2023). Cardamonin as a p38 MAPK signaling pathway activator inhibits human coronavirus OC43 infection in human lung cells. Nutrients 15, 1335. 10.3390/nu15061335 36986065 PMC10057051

[B27] Kenari F. Molnár S. Perjési P. (2021). Reaction of chalcones with cellular thiols. The effect of the 4-Substitution of chalcones and protonation state of the thiols on the addition process. Diastereoselective thiol addition. Molecules 26, 4332. 10.3390/molecules26144332 34299607 PMC8308006

[B28] Klebe G. (2000). Recent developments in structure-based drug design. J. Mol. Med. 78, 269–281. 10.1007/s001090000084 10954199

[B29] Klebe G. Abraham U. Mietzner T. (1994). Molecular similarity indices in a comparative analysis (CoMSIA) of drug molecules to correlate and predict their biological activity. J. Med. Chem. 37, 4130–4146. 10.1021/jm00050a010 7990113

[B30] Kuete V. Mbaveng A. T. Zeino M. Fozing C. D. Ngameni B. Kapche G. D. W. F. (2015). Cytotoxicity of three naturally occurring flavonoid derived compounds (artocarpesin, cycloartocarpesin and isobavachalcone) towards multi-factorial drug-resistant cancer cells. Phytomedicine 22, 1096–1102. 10.1016/j.phymed.2015.07.006 26547532

[B31] Kumar A. Akanksha (2007). Zirconium chloride catalyzed efficient synthesis of 1,3-diaryl-2-propenones in solvent free conditions *via* aldol condensation. J. Mol. Catal. A Chem. 274, 212–216. 10.1016/j.molcata.2007.05.016

[B32] Leon-Gonzalez A. Acero N. Munoz-Mingarro D. Navarro I. Martin-Cordero C. (2015). Chalcones as promising lead compounds on cancer therapy. Curr. Med. Chem. 22, 3407–3425. 10.2174/0929867322666150729114829 26219392

[B33] Li J. Zheng L. Yan M. Wu J. Liu Y. Tian X. (2019). Activity and mechanism of flavokawain A in inhibiting permeability-glycoprotein expression in paclitaxel resistance of lung cancer. Oncol. Lett. 19, 379–387. 10.3892/ol.2019.11069 31897150 PMC6923923

[B34] Liu W. He M. Li Y. Peng Z. Wang G. (2022). A review on synthetic chalcone derivatives as tubulin polymerisation inhibitors. J. Enzyme Inhib. Med. Chem. 37, 9–38. 10.1080/14756366.2021.1976772 34894980 PMC8667932

[B35] Lorca M. Muscia G. C. Pérez-Benavente S. Bautista J. M. Acosta A. González C. (2024). 2D/3D-QSAR model development based on a quinoline pharmacophoric core for the inhibition of plasmodium falciparum: an *in silico* approach with experimental validation. Pharmaceuticals 17, 889. 10.3390/ph17070889 39065740 PMC11279914

[B36] Luczywo A. Soto M. Muscia G. C. Romanelli G. P. Sathicq G. González C. (2023). QSAR‐Guided study for the microwave‐assisted synthesis of 4‐Methylquinoline derivatives with antimycobacterial activity. ChemistrySelect 8, e202300042. 10.1002/slct.202300042

[B37] Ludovico G. S. Barros I. H. S. Sallum L. O. Lima R. S. Valverde C. Camargo A. J. (2021). A new isostructural halogenated chalcone with optical properties. J. Mol. Model. 27, 52. 10.1007/s00894-021-04673-9 33502611

[B38] Marchetti C. Muzii L. Romito A. Benedetti Panici P. (2019). First-line treatment of women with advanced ovarian cancer: focus on bevacizumab. Onco. Targets. Ther. 12, 1095–1103. 10.2147/OTT.S155425 30799939 PMC6371937

[B39] Mellado M. Madrid A. Reyna M. Weinstein-Oppenheimer C. Mella J. Salas C. O. (2018). Synthesis of chalcones with antiproliferative activity on the SH-SY5Y neuroblastoma cell line: quantitative structure–activity relationship models. Med. Chem. Res. 27, 2414–2425. 10.1007/s00044-018-2245-2

[B40] Mellado M. Espinoza L. Madrid A. Mella J. Chávez-Weisser E. Diaz K. (2020). Design, synthesis, antifungal activity, and structure–activity relationship studies of chalcones and hybrid dihydrochromane–chalcones. Mol. Divers. 24, 603–615. 10.1007/s11030-019-09967-y 31161394

[B41] Mellado M. González C. Mella J. Aguilar L. F. Viña D. Uriarte E. (2021). Combined 3D-QSAR and docking analysis for the design and synthesis of chalcones as potent and selective monoamine oxidase B inhibitors. Bioorg. Chem. 108, 104689. 10.1016/j.bioorg.2021.104689 33571810

[B42] Mellado M. Reyna-Jeldes M. Weinstein-Oppenheimer C. Coddou C. Jara-Gutierrez C. Villena J. (2022). Inhibition of Caco-2 and MCF-7 cancer cells using chalcones: synthesis, biological evaluation and computational study. Nat. Prod. Res. 36, 4404–4410. 10.1080/14786419.2021.1984465 34583595

[B43] Michalkova R. Kello M. Kudlickova Z. Gazdova M. Mirossay L. Mojzisova G. (2022). Programmed cell death alterations mediated by synthetic indole chalcone resulted in cell cycle arrest, DNA damage, apoptosis and signaling pathway modulations in breast cancer model. Pharmaceutics 14, 503. 10.3390/pharmaceutics14030503 35335879 PMC8953149

[B44] Michalkova R. Mirossay L. Kello M. Mojzisova G. Baloghova J. Podracka A. (2023). Anticancer potential of natural chalcones: *in vitro* and *in vivo* evidence. Int. J. Mol. Sci. 24, 10354. 10.3390/ijms241210354 37373500 PMC10299153

[B45] Mirzaei S. Hadizadeh F. Eisvand F. Mosaffa F. Ghodsi R. (2020). Synthesis, structure-activity relationship and molecular docking studies of novel quinoline-chalcone hybrids as potential anticancer agents and tubulin inhibitors. J. Mol. Struct. 1202, 127310. 10.1016/j.molstruc.2019.127310

[B46] Momenimovahed Z. Tiznobaik A. Taheri S. Salehiniya H. (2019). Ovarian cancer in the world: epidemiology and risk factors. Int. J. Womens. Health 11, 287–299. 10.2147/IJWH.S197604 31118829 PMC6500433

[B47] Narender T. Papi Reddy K. (2007). A simple and highly efficient method for the synthesis of chalcones by using borontrifluoride-etherate. Tetrahedron Lett. 48, 3177–3180. 10.1016/j.tetlet.2007.03.054

[B48] Nishimura R. Tabata K. Arakawa M. Ito Y. Kimura Y. Akihisa T. (2007). Isobavachalcone, a chalcone constituent of Angelica keiskei, induces apoptosis in neuroblastoma. Biol. Pharm. Bull. 30, 1878–1883. 10.1248/bpb.30.1878 17917255

[B49] Organic Molecules (2004). in Infrared spectroscopy: fundamentals and applications (John Wiley and Sons, Ltd), 71–93. 10.1002/0470011149.ch4

[B50] Ouma R. B. O. Ngari S. M. Kibet J. K. (2024). A review of the current trends in computational approaches in drug design and metabolism. Discov. Public Heal. 21, 108. 10.1186/s12982-024-00229-3

[B51] Ovarian_cancer_Statistics (2025). No title. World Cancer Res. Fund. Available online at: https://www.wcrf.org/preventing-cancer/cancer-statistics/ovarian-cancer-statistics/ (Accessed May 15, 2025).

[B52] Padhye S. Ahmad A. Oswal N. Dandawate P. Rub R. A. Deshpande J. (2010). Fluorinated 2′-hydroxychalcones as garcinol analogs with enhanced antioxidant and anticancer activities. Bioorg. Med. Chem. Lett. 20, 5818–5821. 10.1016/j.bmcl.2010.07.128 20729081

[B53] Pan Q. Yang H. Du Z. Ni Z. Zhu Q. Tu S. (2024). Synthesis, characterization, and anticancer activity of syringaldehyde-derived chalcones against female cancers. Med. Chem. Res. 33, 532–547. 10.1007/s00044-024-03195-2

[B54] Pawlak A. Henklewska M. Hernández Suárez B. Łużny M. Kozłowska E. Obmińska-Mrukowicz B. (2020). Chalcone methoxy derivatives exhibit antiproliferative and proapoptotic activity on canine lymphoma and leukemia cells. Molecules 25, 4362. 10.3390/molecules25194362 32977440 PMC7582533

[B55] Qi Z. Liu M. Liu Y. Zhang M. Yang G. (2014). Tetramethoxychalcone, a chalcone derivative, suppresses proliferation, blocks cell cycle progression, and induces apoptosis of human ovarian cancer cells. PLoS One 9, e106206. 10.1371/journal.pone.0106206 25180593 PMC4152132

[B56] Rautio J. Kumpulainen H. Heimbach T. Oliyai R. Oh D. Järvinen T. (2008). Prodrugs: design and clinical applications. Nat. Rev. Drug Discov. 7, 255–270. 10.1038/nrd2468 18219308

[B57] Ren B. Ablise M. Yang X. Liao B. Yang Z. (2017). Synthesis and biological evaluation of α-methyl-chalcone for anti-cervical cancer activity. Med. Chem. Res. 26, 1871–1883. 10.1007/s00044-017-1891-0

[B58] Rojo de la Vega M. Chapman E. Zhang D. D. (2018). NRF2 and the hallmarks of cancer. Cancer Cell 34, 21–43. 10.1016/j.ccell.2018.03.022 29731393 PMC6039250

[B59] Roy K. Chakraborty P. Mitra I. Ojha P. K. Kar S. Das R. N. (2013). Some case studies on application of “ r m 2 ” metrics for judging quality of quantitative structure–activity relationship predictions: emphasis on scaling of response data. J. Comput. Chem. 34, 1071–1082. 10.1002/jcc.23231 23299630

[B60] Rudrapal M. Khan J. Dukhyil A. A. B. Alarousy R. M. I. I. Attah E. I. Sharma T. (2021). Chalcone scaffolds, bioprecursors of flavonoids: chemistry, bioactivities, and pharmacokinetics. Molecules 26, 7177. 10.3390/molecules26237177 34885754 PMC8659147

[B61] Salanci Š. Vilková M. Martinez L. Mirossay L. Michalková R. Mojžiš J. (2024). The induction of G2/M phase cell cycle arrest and apoptosis by the chalcone derivative 1C in sensitive and resistant ovarian cancer cells is associated with ROS generation. Int. J. Mol. Sci. 25, 7541. 10.3390/ijms25147541 39062784 PMC11277160

[B62] Shin S. Y. Jung H. Ahn S. Hwang D. Yoon H. Hyun J. (2014). Polyphenols bearing cinnamaldehyde scaffold showing cell growth inhibitory effects on the cisplatin-resistant A2780/Cis ovarian cancer cells. Bioorg. Med. Chem. 22, 1809–1820. 10.1016/j.bmc.2014.01.058 24565968

[B63] Sirka L. Doğan H. Bahar M. R. Çalışkan E. Tekin S. Uslu H. (2022). (E)-1-(4-Hydroxyphenyl)-3-(substituted-phenyl) prop-2-en-1-ones: synthesis, *in vitro* cytotoxic activity and molecular docking studies. Acta Chim. Slov. 69, 281–292. 10.17344/acsi.2021.7080 35861092

[B64] Sliwoski G. Kothiwale S. Meiler J. Lowe E. W. (2014). Computational methods in drug discovery. Pharmacol. Rev. 66, 334–395. 10.1124/pr.112.007336 24381236 PMC3880464

[B65] Srilaxmi D. Sreenivasulu R. Mak K.-K. Pichika M. R. Jadav S. S. Ahsan M. J. (2021). Design, synthesis, anticancer evaluation and molecular docking studies of chalcone linked pyrido[4,3-b]pyrazin-5(6H)-one derivatives. J. Mol. Struct. 1229, 129851. 10.1016/j.molstruc.2020.129851

[B66] Takac P. Kello M. Pilatova M. B. Kudlickova Z. Vilkova M. Slepcikova P. (2018). New chalcone derivative exhibits antiproliferative potential by inducing G2/M cell cycle arrest, mitochondrial-mediated apoptosis and modulation of MAPK signalling pathway. Chem. Biol. Interact. 292, 37–49. 10.1016/j.cbi.2018.07.005 29981726

[B67] Traverso N. Ricciarelli R. Nitti M. Marengo B. Furfaro A. L. Pronzato M. A. (2013). Role of glutathione in cancer progression and chemoresistance. Oxid. Med. Cell. Longev. 2013, 1–10. 10.1155/2013/972913 23766865 PMC3673338

[B68] Tropsha A. (2010). Best practices for QSAR model development, validation, and exploitation. Mol. Inf. 29, 476–488. 10.1002/minf.201000061 27463326

[B69] Urniaż R. D. Jóźwiak K. (2013). X-ray crystallographic structures as a source of ligand alignment in 3D-QSAR. J. Chem. Inf. Model. 53, 1406–1414. 10.1021/ci400004e 23705769

[B70] Valenzuela M. Glorieux C. Stockis J. Sid B. Sandoval J. M. Felipe K. B. (2014). Retinoic acid synergizes ATO-Mediated cytotoxicity by precluding Nrf2 activity in AML cells. Br. J. Cancer 111, 874–882. 10.1038/bjc.2014.380 25003661 PMC4150280

[B71] Wang L. Wang X. Zhu X. Zhong L. Jiang Q. Wang Y. (2024). Drug resistance in ovarian cancer: from mechanism to clinical trial. Mol. Cancer 23, 66. 10.1186/s12943-024-01967-3 38539161 PMC10976737

[B72] Wilkes J. G. Stoyanova-Slavova I. B. Buzatu D. A. (2016). Alignment-independent technique for 3D QSAR analysis. J. Comput. Aided. Mol. Des. 30, 331–345. 10.1007/s10822-016-9909-0 27026022 PMC4833814

[B73] Yamali C. Gul H. I. Sakagami H. Supuran C. T. (2016). Synthesis and bioactivities of halogen bearing phenolic chalcones and their corresponding bis mannich bases. J. Enzyme Inhib. Med. Chem. 31, 125–131. 10.1080/14756366.2016.1221825 27594305

[B74] Yan M. Wang H. Wei R. Li W. (2024). Arsenic trioxide: applications, mechanisms of action, toxicity and rescue strategies to date. Arch. Pharm. Res. 47, 249–271. 10.1007/s12272-023-01481-y 38147202

[B75] Zhang D. D. (2025). Thirty years of NRF2: advances and therapeutic challenges. Nat. Rev. Drug Discov. 24, 421–444. 10.1038/s41573-025-01145-0 40038406

[B76] Zhang S. Li T. Zhang Y. Xu H. Li Y. Zi X. (2016). A new brominated chalcone derivative suppresses the growth of gastric cancer cells *in vitro* and *in vivo* involving ROS mediated up-regulation of DR5 and 4 expression and apoptosis. Toxicol. Appl. Pharmacol. 309, 77–86. 10.1016/j.taap.2016.08.023 27594528 PMC5507202

