# Supplementary material for: 3D-QSAR study for the development of chalcone-based inhibitors targeting ovarian cancer cells with experimental validation
Source: Front Pharmacol. 2026 Mar 9;17:1746658. doi: 10.3389/fphar.2026.1746658 (PMC13006584; doi:10.3389/fphar.2026.1746658)
Supplement: Supplementary file 1 [file DataSheet1.docx]

**SUPPLEMENTARY MATERIALS**

**3D-QSAR study for the development of chalcone-based inhibitors targeting ovarian cancer cells with experimental validation**

Manuel Valenzuela-Valderrama^1^, Aranxa Varas^2^, Mariaignacia Rubilar^1^, Marcos Lorca^3^, Jaime Mella^4,5^, Christian Espinosa-Bustos^6^, Marco Mellado^7,8^*, and Javier Echeverría^9^*.

^1^ Laboratorio de Carcinogénesis Molecular, Facultad de Medicina y Ciencias de la Salud, Universidad Central de Chile, Santiago, Chile.

^2^ Carrera de Tecnología Médica, Facultad de Medicina y Ciencias de la Salud, Universidad Central de Chile, Santiago, Chile.

^3^ Facultad de Ciencias de la Vida, Carrera de Química y Farmacia, Universidad Viña del Mar, Viña del Mar, Chile.

^4^ Instituto de Química, Facultad de Ciencias, Universidad de Valparaíso, Valparaíso, Chile.

^5^ Centro de Investigación, Desarrollo e Innovación de Productos Bioactivos (CInBIO), Universidad de Valparaíso, Valparaíso, Chile.

^6^ Departamento de Farmacia, Facultad de Química y de Farmacia, Pontificia Universidad Católica de Chile, Santiago, Chile.

^7^ Dirección de Investigación, Universidad Bernardo O’Higgins, Santiago, Chile.

^8^ Centro de Investigación en Ingeniería de Materiales, Universidad Central de Chile, Santiago, Chile.

^9^ Departamento de Ciencias del Ambiente, Facultad de Química y Biología, Universidad de Santiago de Chile, Santiago, Chile.

Correspondence: Dr. Marco Mellado, Email: marcomellado86@gmail.com; Dr. Javier Echeverria, Email: javier.echeverriam@usach.cl

**Table of contents**

1. **Information on theoretical models** pages 3-15
2. **Spectroscopic evidence of the synthetic compounds 65-76** pages 16-51
3. **Original Images of Biological Assessment** pages 52-56


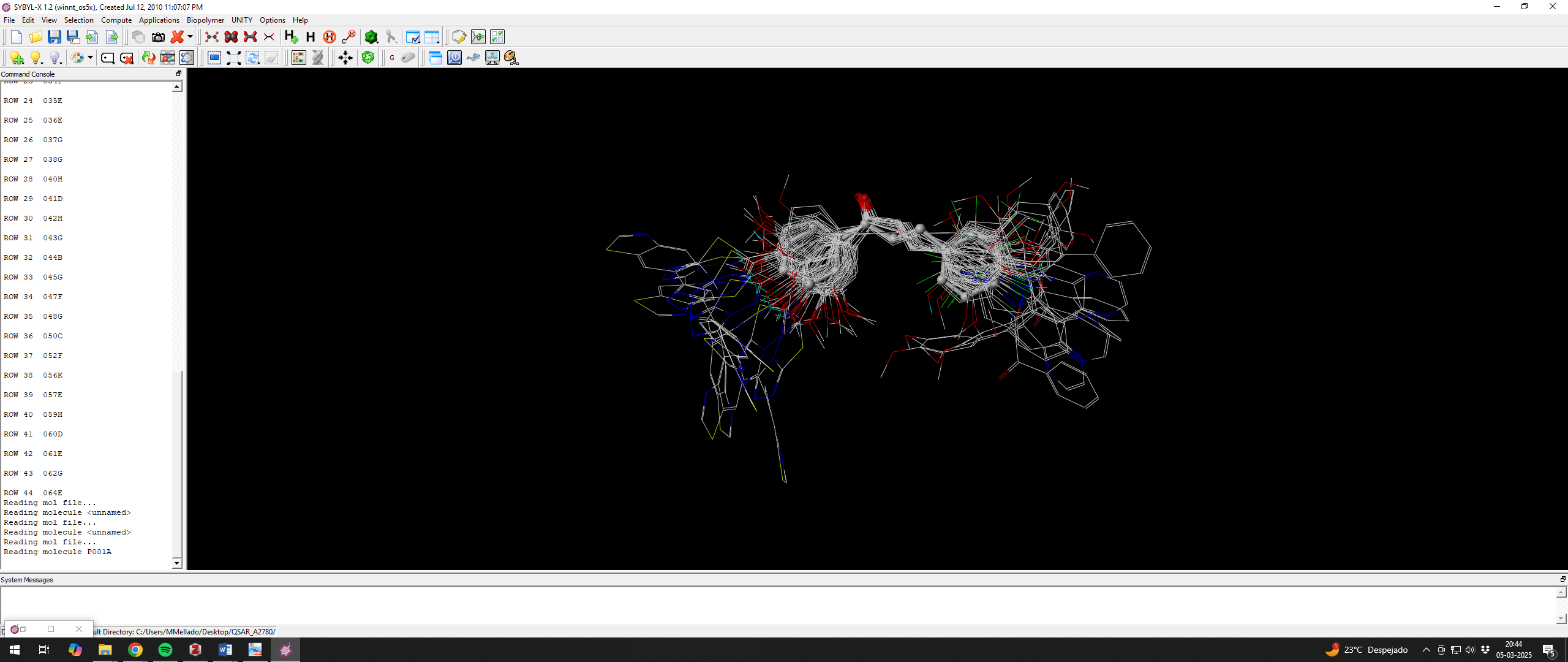


**Figure S1.** The superimposed structures of all compounds used in the 3D-QSAR model.

**Figure S2.** Histogram of frequency distribution data

**Table S1.** Chemical structure of the chalcones used in the 3D-QSAR study.

|   **001**  (Mirzaei et al., 2020) |   **002**  (Mirzaei et al., 2020) |   **003**  (Mirzaei et al., 2020) |
| --- | --- | --- |
|   **004**  (Mirzaei et al., 2020) |   **005**  (Mirzaei et al., 2020) |   **006**  (Mirzaei et al., 2020) |
|   **007**  (Mirzaei et al., 2020) |   **008**  (Mirzaei et al., 2020) |   **009**  (Mirzaei et al., 2020) |
|   **010**  (Srilaxmi et al., 2021) |   **011**  (Srilaxmi et al., 2021) |   **012**  (Srilaxmi et al., 2021) |
|   **013**  (Srilaxmi et al., 2021) |   **014**  (Srilaxmi et al., 2021) |   **015**  (Srilaxmi et al., 2021) |

|   **016**  (Srilaxmi et al., 2021) |   **017**  (Srilaxmi et al., 2021) |   **018**  (Srilaxmi et al., 2021) |
| --- | --- | --- |
|   **019**  (Srilaxmi et al., 2021) |   **020**  (Sirka et al., 2022) |   **021**  (Sirka et al., 2022) |
|   **022**  (Sirka et al., 2022) |   **023**  (Sirka et al., 2022) |   **024**  (Sirka et al., 2022) |
|   **025**  (Sirka et al., 2022) |   **026**  (Sirka et al., 2022) |   **027**  (Sirka et al., 2022) |
|   **028**  (Sirka et al., 2022) |   **029**  (Sirka et al., 2022) |  **030**  (Qi et al., 2014) |
|   **031**  (Bujji et al., 2020) |   **032**  (Bujji et al., 2020) |   **033**  (Bujji et al., 2020) |

|   **034**  (Bujji et al., 2020) |   **035**  (Bujji et al., 2020) |   **036**  (Bujji et al., 2020) |
| --- | --- | --- |
|   **037**  (Bujji et al., 2020) |   **038**  (Bujji et al., 2020) |   **039**  (Bujji et al., 2020) |
|   **040**  (Bujji et al., 2020) |   **041**  (Pan et al., 2024) |   **042**  (Pan et al., 2024) |
|   **043**  (Pan et al., 2024) |   **044**  (Pan et al., 2024) |   **045**  (Pan et al., 2024) |
|   **046**  (Pan et al., 2024) |   **047**  (Pan et al., 2024) |   **048**  (Pan et al., 2024) |
|   **049**  (Pan et al., 2024) |   **050**  (Pan et al., 2024) |   **051**  (Pan et al., 2024) |
|   **052**  (Pan et al., 2024) |   **053**  (Pan et al., 2024) |   **054**  (Pan et al., 2024) |

|   **055**  (Pan et al., 2024) |   **056**  (Pan et al., 2024) |   **057**  (Pan et al., 2024) |
| --- | --- | --- |
|   **058**  (Pan et al., 2024) |   **059**  (Pan et al., 2024) |   **060**  (Pan et al., 2024) |
|   **061**  (Pan et al., 2024) |   **062**  (Pan et al., 2024) |   **063**  (Pan et al., 2024) |
|   **064**  (Pan et al., 2024) |  |  |

**Table S2.** Statistical parameters and field combinations for CoMFA and CoMSIA.

| **Field** | **q^2^** | **N** | **SEP** | **SEE** | **r^2^_ncv_** | **F** | **Field** | | | | |
| --- | --- | --- | --- | --- | --- | --- | --- | --- | --- | --- | --- |
|  |  |  |  |  |  |  | **S** | **E** | **H** | **D** | **A** |
| ***CoMFA*** |  |  |  |  |  |  |  |  |  |  |  |
| S | 0.674 | 3 | 0.331 | 0.123 | 0.955 | 285.2 | 1 |  |  |  |  |
| E | 0.596 | 5 | 0.378 | 0.216 | 0.869 | 50.4 |  | 1 |  |  |  |
| **SE** | **0.763** | **3** | **0.304** | **0.147** | **0.936** | **195.3** | **0.633** | **0.367** |  |  |  |
| ***CoMSIA*** |  |  |  |  |  |  |  |  |  |  |  |
| S | 0.778 | 6 | 0.284 | 0.091 | 0.977 | 266.3 | 1 |  |  |  |  |
| E | 0.575 | 5 | 0.388 | 0.174 | 0.915 | 81.3 |  | 1 |  |  |  |
| H | 0.434 | 11 | 0.488 | 0.027 | 0.998 | 1718 |  |  | 1 |  |  |
| D | 0.137 | 1 | 0.526 | 0.494 | 0.238 | 13.1 |  |  |  | 1 |  |
| A | 0.232 | 1 | 0.496 | 0.437 | 0.403 | 28.3 |  |  |  |  | 1 |
| SE | 0.808 | 5 | 0.261 | 0.106 | 0.968 | 231.3 | 0.497 | 0.503 |  |  |  |
| SEH | 0.776 | 4 | 0.278 | 0.1 | 0.971 | 324.7 | 0.331 | 0.38 | 0.29 |  |  |
| SEHD | 0.713 | 4 | 0.315 | 0.106 | 0.967 | 290 | 0.275 | 0.331 | 0.223 | 0.171 |  |
| SEHA | 0.778 | 4 | 0.277 | 0.104 | 0.969 | 302.4 | 0.274 | 0.36 | 0.243 |  | 0.122 |
| SED | 0.748 | 4 | 0.295 | 0.127 | 0.953 | 199.8 | 0.389 | 0.398 |  | 0.214 |  |
| **SEA** | **0.789** | **3** | **0.267** | **0.164** | **0.92** | **152.7** | **0.368** | **0.443** |  |  | **0.189** |
| SEDA | 0.769 | 4 | 0.304 | 0.119 | 0.959 | 228.1 | 0.282 | 0.364 |  | 0.223 | 0.131 |
| SH | 0.723 | 12 | 0.347 | 0.011 | 1 | 9337.2 | 0.528 |  | 0.472 |  |  |
| SD | 0.651 | 4 | 0.347 | 0.177 | 0.909 | 97.2 | 0.727 |  |  | 0.273 |  |
| SA | 0.716 | 20 | 0.408 | 0.006 | 1 | 20261.5 | 0.775 |  |  |  | 0.225 |
| SHD | 0.62 | 14 | 0.42 | 0.004 | 1 | 57541.8 | 0.402 |  | 0.358 | 0.24 |  |
| SHA | 0.693 | 5 | 0.35 | 0.083 | 0.98 | 381.1 | 0.473 |  | 0.386 |  | 0.141 |
| SDA | 0.647 | 4 | 0.349 | 0.172 | 0.914 | 104 | 0.604 |  |  | 0.245 | 0.151 |
| SHDA | 0.611 | 15 | 0.433 | 0.004 | 1 | 70218.5 | 0.364 |  | 0.323 | 0.214 | 0.099 |
| EH | 0.623 | 5 | 0.365 | 0.102 | 0.97 | 249.5 |  | 0.463 | 0.537 |  |  |
| ED | 0.52 | 2 | 0.397 | 0.316 | 0.696 | 46.8 |  | 0.615 |  | 0.385 |  |
| EA | 0.69 | 3 | 0.323 | 0.219 | 0.857 | 79.8 |  | 0.613 |  |  | 0.387 |
| EHD | 0.519 | 1 | 0.393 | 0.348 | 0.621 | 68.9 |  | 0.556 | 0.152 | 0.292 |  |
| EHA | 0.701 | 5 | 0.326 | 0.093 | 0.976 | 304.5 |  | 0.405 | 0.386 |  | 0.209 |
| EDA | 0.746 | 11 | 0.327 | 0.037 | 0.997 | 882.4 |  | 0.454 |  | 0.328 | 0.218 |
| EHDA | 0.684 | 14 | 0.383 | 0.005 | 1 | 33319.4 |  | 0.344 | 0.274 | 0.223 | 0.16 |
| HD | 0.258 | 13 | 0.577 | 0.02 | 0.999 | 2536.8 |  |  | 0.701 | 0.299 |  |
| HA | 0.388 | 7 | 0.478 | 0.071 | 0.987 | 376.4 |  |  | 0.751 |  | 0.249 |
| HDA | 0.27 | 17 | 0.615 | 0.007 | 1 | 18222 |  |  | 0.592 | 0.242 | 0.166 |
| DA | 0.178 | 1 | 0.513 | 0.473 | 0.303 | 18.2 |  |  |  | 0.738 | 0.262 |
| ALL | 0.745 | 4 | 0.297 | 0.104 | 0.969 | 301.3 | 0.227 | 0.317 | 0.183 | 0.17 | 0.103 |

*q^2^*, the square of the LOO cross-validation (CV) coefficient; N, the optimum number of components; SEP, standard error of prediction; SEE, standard error of estimation of non CV analysis; *r^2^_ncv_*, square of the non CV coefficient; F, F-test value; S, E, H, D and A are the steric, electrostatic, hydrophobic, hydrogen-bond donor, and hydrogen-bond acceptor contributions respectively.

**Table S3.** Summary of the CoMFA-SE and CoMSIA-SEA models developed for ovarian cancer cell line A2780.

| **Field** | **q^2^** | **N** | **SEP** | **SEE** | **r^2^_ncv_** | **F** | **SSD** | **PRESS** | **r^2^_pred_** |
| --- | --- | --- | --- | --- | --- | --- | --- | --- | --- |
| CoMFA-SE | 0.763 | 3 | 0.304 | 0.147 | 0.936 | 195.3 | 0.869 | 2.785 | 0.688 |
| CoMSIA-SEA | 0.789 | 3 | 0.267 | 0.164 | 0.920 | 152.7 | 1.103 | 3.176 | 0.653 |

*q^2^*, the square of the LOO cross-validation (CV) coefficient; N, the optimum number of components; SEP, standard error of prediction; SEE, standard error of estimation of non CV analysis; *r^2^_ncv_*, square of the non CV coefficient; F, F-test value; SSD, is the value calculated according to the equation 8; PRESS, is the value calculated according to the equation 7; r^2^_pred_, is the r^2^ value of predictive for the test set.

**Table S4.** Experimental pIC_50_, predicted pIC_50,_ and residual values for the analyzed compounds obtained with the CoMFA and CoMSIA models.

| **Compound** | **pIC_50_** | | | | |
| --- | --- | --- | --- | --- | --- |
|  | **Experimental** | **CoMFA** | **Residual** | **CoMSIA** | **Residual** |
| **001 ^a^** | 4.138 | 4.326 | -0.188 | 4.389 | -0.251 |
| **002 ^a^** | 4.660 | 4.464 | 0.196 | 4.432 | 0.228 |
| **003 ^a^** | 4.424 | 4.525 | -0.101 | 4.464 | -0.040 |
| **004 ^a^** | 4.859 | 4.830 | 0.029 | 4.747 | 0.112 |
| **005 ^b^** | 4.827 | 4.885 | -0.058 | 4.748 | 0.080 |
| **006 ^a^** | 5.506 | 5.480 | 0.026 | 5.444 | 0.062 |
| **007 ^a^** | 4.254 | 4.330 | -0.076 | 4.454 | -0.200 |
| **008 ^a^** | 4.551 | 4.664 | -0.113 | 4.556 | -0.005 |
| **009 ^b^** | 5.635 | 5.286 | 0.348 | 5.112 | 0.522 |
| **010 ^b^** | 5.334 | 5.101 | 0.233 | 5.168 | 0.167 |
| **011 ^a^** | 6.009 | 5.907 | 0.102 | 5.977 | 0.032 |
| **012 ^a^** | 5.879 | 6.042 | -0.163 | 6.059 | -0.180 |
| **013 ^b^** | 5.633 | 5.716 | -0.083 | 5.530 | 0.103 |
| **014 ^b^** | 5.271 | 5.302 | -0.032 | 5.254 | 0.017 |
| **015 ^b^** | 4.870 | 4.845 | 0.025 | 4.861 | 0.009 |
| **016 ^a^** | 4.894 | 4.933 | -0.039 | 4.915 | -0.021 |
| **017 ^c^** | 7.921 | 5.728 | 2.193 | 6.054 | 1.867 |
| **018 ^c^** | 6.745 | 5.473 | 1.272 | 5.362 | 1.383 |
| **019 ^b^** | 5.075 | 5.077 | -0.002 | 5.061 | 0.013 |
| **020 ^a^** | 4.963 | 5.159 | -0.196 | 5.135 | -0.172 |
| **021 ^a^** | 4.089 | 4.382 | -0.293 | 4.551 | -0.462 |
| **022 ^a^** | 4.899 | 4.972 | -0.073 | 4.912 | -0.013 |
| **023 ^a^** | 4.288 | 4.263 | 0.025 | 4.397 | -0.109 |
| **024 ^a^** | 4.182 | 4.473 | -0.291 | 4.482 | -0.300 |
| **025 ^a^** | 5.161 | 5.219 | -0.058 | 5.068 | 0.093 |
| **026 ^a^** | 5.011 | 4.928 | 0.083 | 4.982 | 0.029 |
| **027 ^a^** | 5.215 | 5.161 | 0.054 | 5.099 | 0.116 |
| **028 ^a^** | 4.847 | 4.541 | 0.306 | 4.673 | 0.174 |
| **029 ^a^** | 4.973 | 4.772 | 0.201 | 4.784 | 0.189 |
| **030 ^b^** | 5.456 | 5.157 | 0.299 | 5.055 | 0.401 |
| **031 ^a^** | 5.028 | 5.116 | -0.088 | 5.190 | -0.162 |
| **032 ^a^** | 5.755 | 5.715 | 0.040 | 5.817 | -0.062 |
| **033 ^b^** | 5.635 | 5.712 | -0.078 | 5.626 | 0.008 |
| **034 ^a^** | 6.222 | 6.254 | -0.032 | 6.195 | 0.027 |
| **035 ^a^** | 5.721 | 5.577 | 0.144 | 5.661 | 0.060 |
| **036 ^a^** | 5.171 | 5.201 | -0.030 | 5.061 | 0.110 |
| **037 ^a^** | 5.914 | 5.982 | -0.068 | 5.867 | 0.047 |
| **038 ^a^** | 6.523 | 6.356 | 0.167 | 6.441 | 0.082 |
| **039 ^b^** | 5.418 | 5.327 | 0.091 | 5.691 | -0.274 |
| **040 ^a^** | 5.613 | 5.773 | -0.160 | 5.813 | -0.200 |
| **041 ^a^** | 5.090 | 5.032 | 0.058 | 5.056 | 0.034 |
| **042 ^a^** | 4.825 | 4.864 | -0.039 | 4.841 | -0.016 |
| **043 ^a^** | 4.768 | 4.698 | 0.070 | 4.662 | 0.106 |
| **044 ^a^** | 4.806 | 4.567 | 0.239 | 4.622 | 0.184 |
| **045 ^a^** | 4.458 | 4.560 | -0.102 | 4.624 | -0.166 |
| **046 ^b^** | 5.418 | 5.124 | 0.294 | 5.024 | 0.394 |
| **047 ^a^** | 5.122 | 5.023 | 0.099 | 4.958 | 0.164 |
| **048 ^a^** | 4.902 | 5.055 | -0.153 | 4.989 | -0.087 |
| **049 ^b^** | 5.199 | 5.017 | 0.182 | 4.837 | 0.362 |
| **050 ^a^** | 4.506 | 4.557 | -0.051 | 4.614 | -0.108 |
| **051 ^b^** | 4.806 | 4.821 | -0.015 | 4.834 | -0.028 |
| **052 ^a^** | 4.658 | 4.794 | -0.136 | 4.673 | -0.015 |
| **053 ^b^** | 4.622 | 4.667 | -0.045 | 4.672 | -0.050 |
| **054 ^b^** | 5.218 | 5.021 | 0.197 | 5.205 | 0.013 |
| **055 ^b^** | 4.811 | 4.805 | 0.006 | 4.934 | -0.123 |
| **056 ^a^** | 4.916 | 4.656 | 0.260 | 4.745 | 0.171 |
| **057 ^a^** | 4.967 | 5.018 | -0.051 | 5.162 | -0.195 |
| **058 ^b^** | 4.185 | 4.789 | -0.604 | 4.648 | -0.463 |
| **059 ^a^** | 4.691 | 4.606 | 0.085 | 4.498 | 0.193 |
| **060 ^a^** | 4.820 | 4.589 | 0.231 | 4.551 | 0.269 |
| **061 ^a^** | 4.666 | 4.637 | 0.029 | 4.642 | 0.024 |
| **062 ^a^** | 5.202 | 5.193 | 0.009 | 4.993 | 0.209 |
| **063 ^b^** | 4.432 | 4.661 | -0.229 | 4.605 | -0.174 |
| **064 ^a^** | 4.712 | 4.661 | 0.051 | 4.662 | 0.050 |

^a^: Compounds used for the training set; ^b^: Compounds used for the test set; ^c^: Outlier compound

**Table S5.** Summary of external validation parameters for CoMFA-SE and CoMSIA-SEA models.

|  | **CoMFA-SE** | | | **CoMSIA-SEA** | | |
| --- | --- | --- | --- | --- | --- | --- |
| **Metric** | **Training Set** | **Test Set including outliers** | **Test Set excluding outliers** | **Training Set** | **Test Set including outliers** | **Test Set excluding outliers** |
| r^2^ | 0.9361 | 0.4277 | 0.7198 | 0.9198 | 0.4901 | 0.6445 |
| CCC | 0.9670 | 0.5372 | 0.8174 | 0.9582 | 0.6067 | 0.7776 |
| q^2^_F1_ | 0.9361 | 0.4277 | 0.7198 | 0.9198 | 0.4901 | 0.6445 |
| q^2^_F2_ | 0.9317 | -2.3598 | 0.4693 | 0.9128 | -1.3091 | 0.3845 |
| q^2^_F3_ | 0.9361 | 0.0855 | 0.6097 | 0.9197 | 0.2084 | 0.6239 |
| MAE | 0.1137 | 0.3143 | 0.1567 | 0.1257 | 0.3225 | 0.1778 |
| RMSD | 0.1399 | 0.6040 | 0.2198 | 0.1567 | 0.5702 | 0.2476 |
| r^2^_0_ | 0.9318 | -1.6536 | 0.4887 | 0.9128 | -0.7470 | 0.4297 |
| r^2^_m_ | 0.8746 | -0.1893 | 0.3738 | 0.8433 | -0.0550 | 0.3458 |
| Δr^2^_m_ | 0.0615 | 0.5924 | 0.2721 | 0.0765 | 0.5103 | 0.2877 |

r²: Coefficient of determination. CCC: Concordance correlation coefficient. q^2^F1: Predictive squared correlation using experimental mean. q^2^F2: Predictive squared correlation using predicted mean. q^2^F3: Predictive squared correlation adjusted for intercept bias. MAE: Mean absolute error. RMSD: Root mean square deviation. r^2^_0_: Squared correlation from regression through the origin. r^2^_m_: Metric R² penalizing systematic bias. Δr^2^_m_ Difference in metric r^2^ between direct and inverse regressions.

**Table S6.** *Y*-random test of CoMFA-SE and CoMSIA-SEA developed.

| **Iteration** | **CoMFA-SE** | | |  | **CoMSIA-SEA** | | |
| --- | --- | --- | --- | --- | --- | --- | --- |
|  | **q^2^** | **N** | **r^2^** |  | **q^2^** | **N** | **r^2^** |
| **Random 1** | 0.095 | 4 | 0.905 |  | 0.076 | 5 | 0.814 |
| **Random 2** | -0.068 | 16 | 1 |  | -0.079 | 4 | 0.77 |
| **Random 3** | -0.047 | 1 | 0.086 |  | -0.078 | 1 | 0.154 |
| **Random 4** | 0.086 | 14 | 1 |  | -0.129 | 1 | 0.235 |
| **Random 5** | -0.11 | 5 | 0.955 |  | -0.113 | 1 | 0.317 |
| **Random 6** | 0.029 | 1 | 0.187 |  | 0.026 | 1 | 0.284 |
| **Random 7** | -0.06 | 1 | 0.078 |  | -0.092 | 20 | 1 |
| **Random 8** | -0.007 | 2 | 0.487 |  | -0.019 | 1 | 0.347 |
| **Random 9** | -0.534 | 1 | 0.238 |  | -0.11 | 3 | 0.715 |
| **Random 10** | -0.343 | 1 | 0.168 |  | -0.329 | 1 | 0.365 |

**References**

Bujji, S., Edigi, P. K., and Subhashini, N. J. P. (2020). Synthesis and evaluation of novel 1,2,4-triazolo-[3,4-b]-1,3,4-thiadiazole tethered chalcone hybrids as potential anticancer agents. *J. Heterocycl. Chem.* 57, 3318–3325. doi: 10.1002/jhet.4047

Mirzaei, S., Hadizadeh, F., Eisvand, F., Mosaffa, F., and Ghodsi, R. (2020). Synthesis, structure-activity relationship and molecular docking studies of novel quinoline-chalcone hybrids as potential anticancer agents and tubulin inhibitors. *J. Mol. Struct.* 1202, 127310. doi: 10.1016/j.molstruc.2019.127310

Pan, Q., Yang, H., Du, Z., Ni, Z., Zhu, Q., Tu, S., et al. (2024). Synthesis, characterization, and anticancer activity of syringaldehyde-derived chalcones against female cancers. *Med. Chem. Res.* 33, 532–547. doi: 10.1007/s00044-024-03195-2

Qi, Z., Liu, M., Liu, Y., Zhang, M., and Yang, G. (2014). Tetramethoxychalcone, a Chalcone Derivative, Suppresses Proliferation, Blocks Cell Cycle Progression, and Induces Apoptosis of Human Ovarian Cancer Cells. *PLoS One* 9, e106206. doi: 10.1371/journal.pone.0106206

Sirka, L., Dogan, H., Bahar, M. R., Caliskan, E., Tekin, S., Uslu, H., et al. (2022). (*E*)-1-(4-Hydroxyphenyl)-3-(substituted-phenyl) prop-2-en-1-ones: Synthesis, *In* *Vitro* Cytotoxic Activity and Molecular Docking Studies. *Acta Chim. Slov.* 69, 281–292. doi: 10.17344/acsi.2021.7080

Srilaxmi, D., Sreenivasulu, R., Mak, K.-K., Pichika, M. R., Jadav, S. S., Ahsan, M. J., et al. (2021). Design, synthesis, anticancer evaluation and molecular docking studies of chalcone linked pyrido[4,3-b]pyrazin-5(6*H*)-one derivatives. *J. Mol. Struct.* 1229, 129851. doi: 10.1016/j.molstruc.2020.129851

**Spectroscopic evidence of the synthetic compounds 65-76**

**Spectrum S1.** FT-IR of the compound (*E*)-3-(3-hydroxyphenyl)-1-phenylprop-2-en-1-one (**065**).


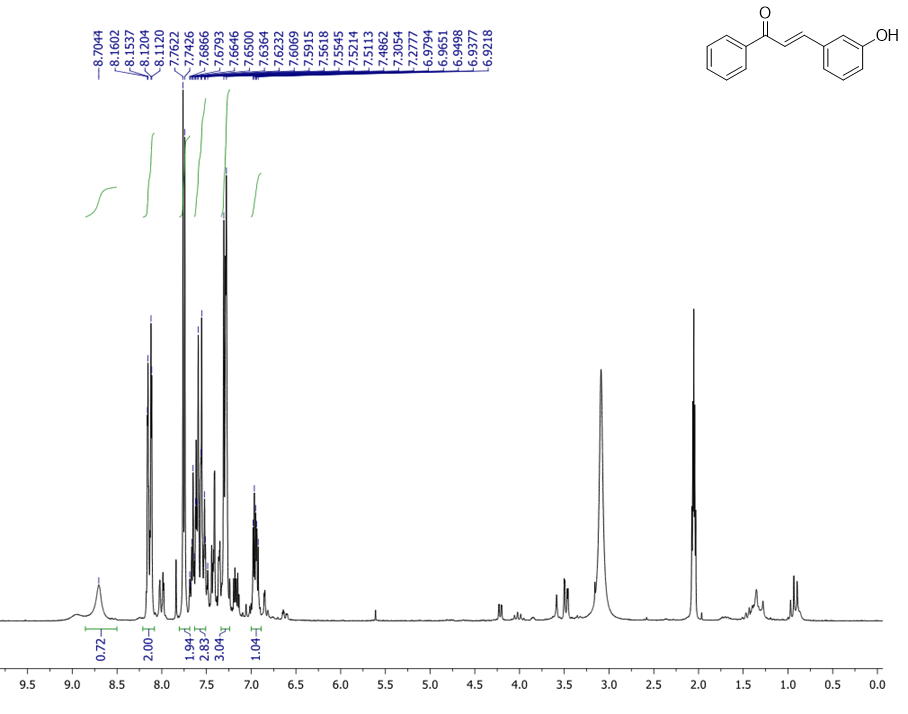


**Spectrum S2.** ^1^H-NMR of the compound (*E*)-3-(3-hydroxyphenyl)-1-phenylprop-2-en-1-one (**065**).


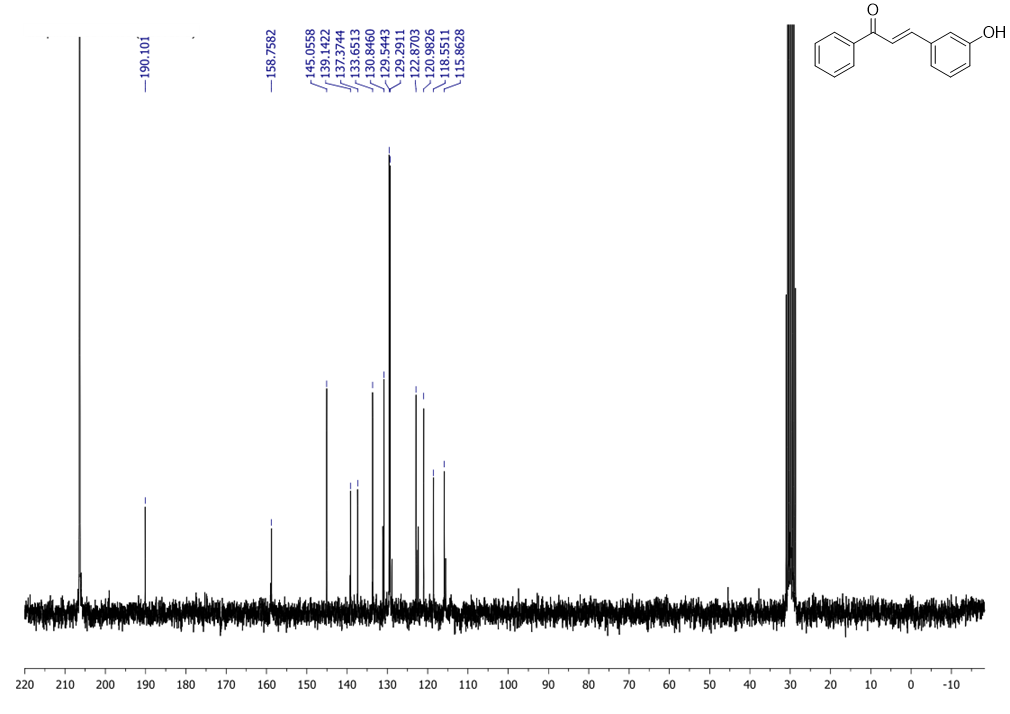


**Spectrum S3.** ^13^C-NMR of the compound (*E*)-3-(3-hydroxyphenyl)-1-phenylprop-2-en-1-one (**065**).

**Spectrum S4.** FT-IR of the compound (*E*)-3-(4-hydroxyphenyl)-1-phenylprop-2-en-1-one (**066**).


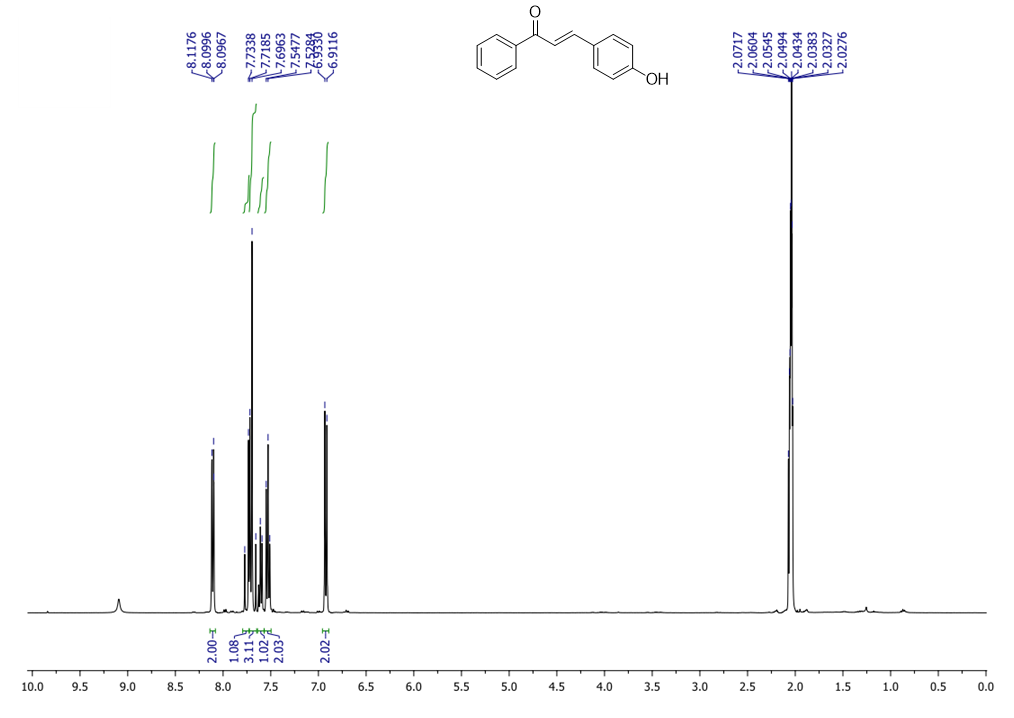


**Spectrum S5.** ^1^H-NMR of the compound (*E*)-3-(4-hydroxyphenyl)-1-phenylprop-2-en-1-one (**066**).


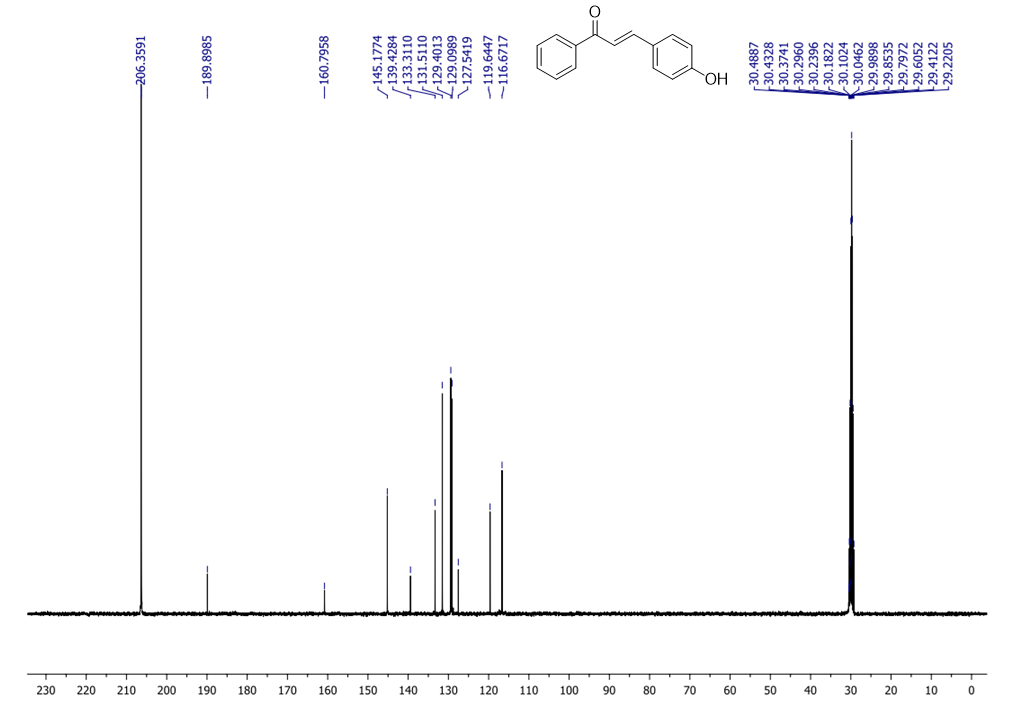


**Spectrum S6.** ^13^C-NMR of the compound (*E*)-3-(4-hydroxyphenyl)-1-phenylprop-2-en-1-one (**066**).

**Spectrum S7.** FT-IR of the compound (*E*)-3-(3-methoxyphenyl)-1-phenylprop-2-en-1-one (**067**).


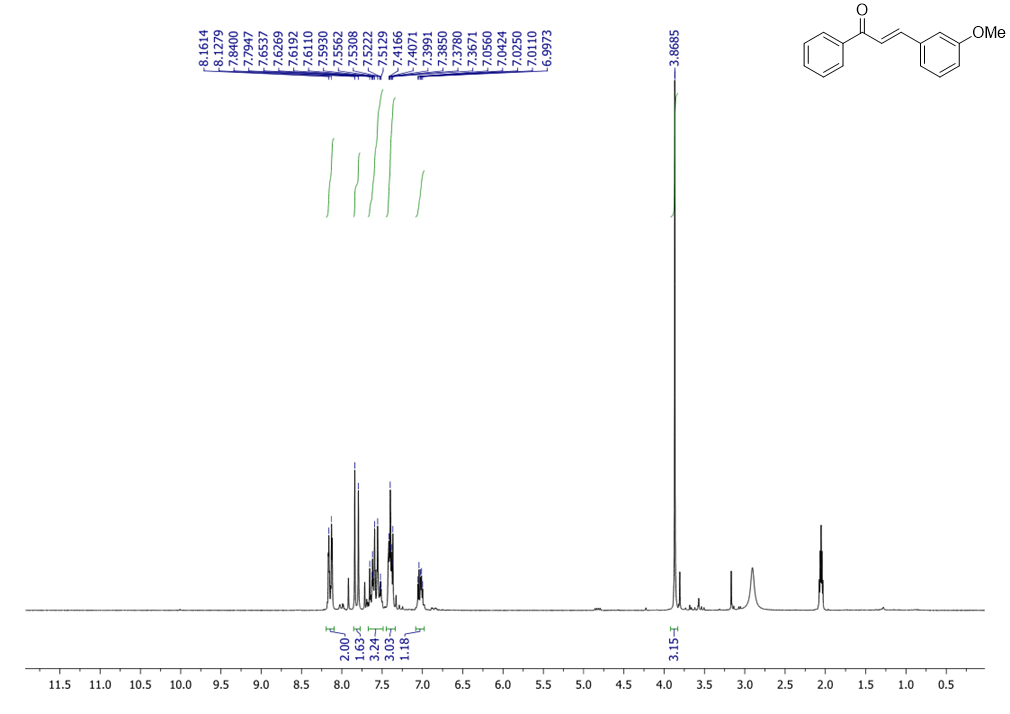


**Spectrum S8.** ^1^H-NMR of the compound (*E*)-3-(3-methoxyphenyl)-1-phenylprop-2-en-1-one (**067**).


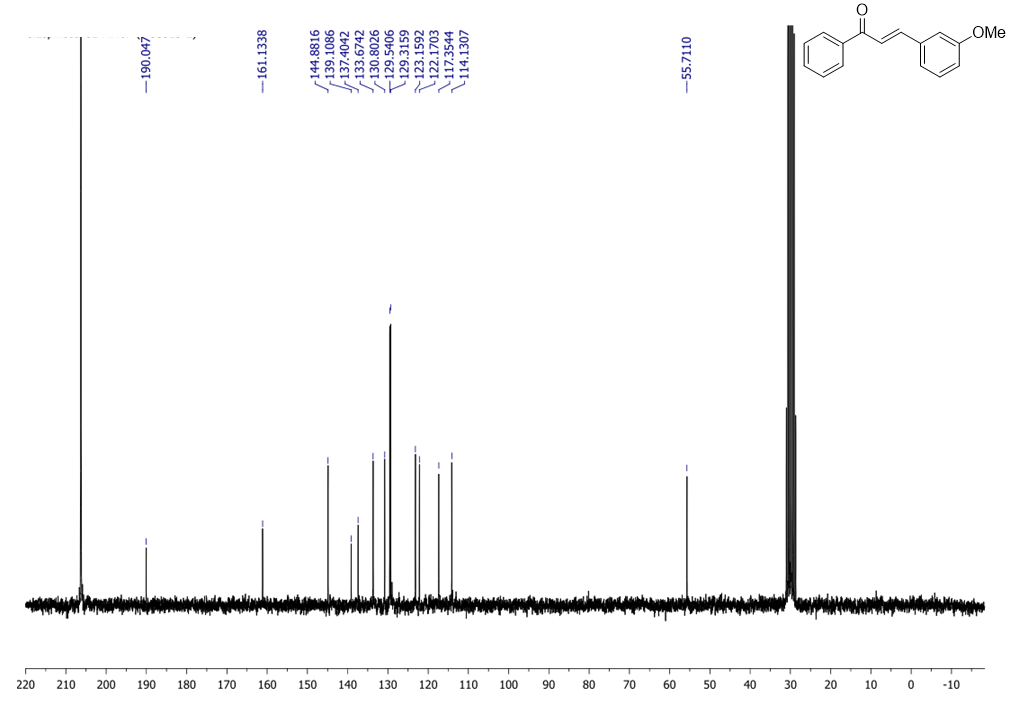


**Spectrum S9.** ^13^C-NMR of the compound (*E*)-3-(3-methoxyphenyl)-1-phenylprop-2-en-1-one (**067**).

**Spectrum S10.** FT-IR of the compound (*E*)-3-(4-methoxyphenyl)-1-phenylprop-2-en-1-one (**068**).


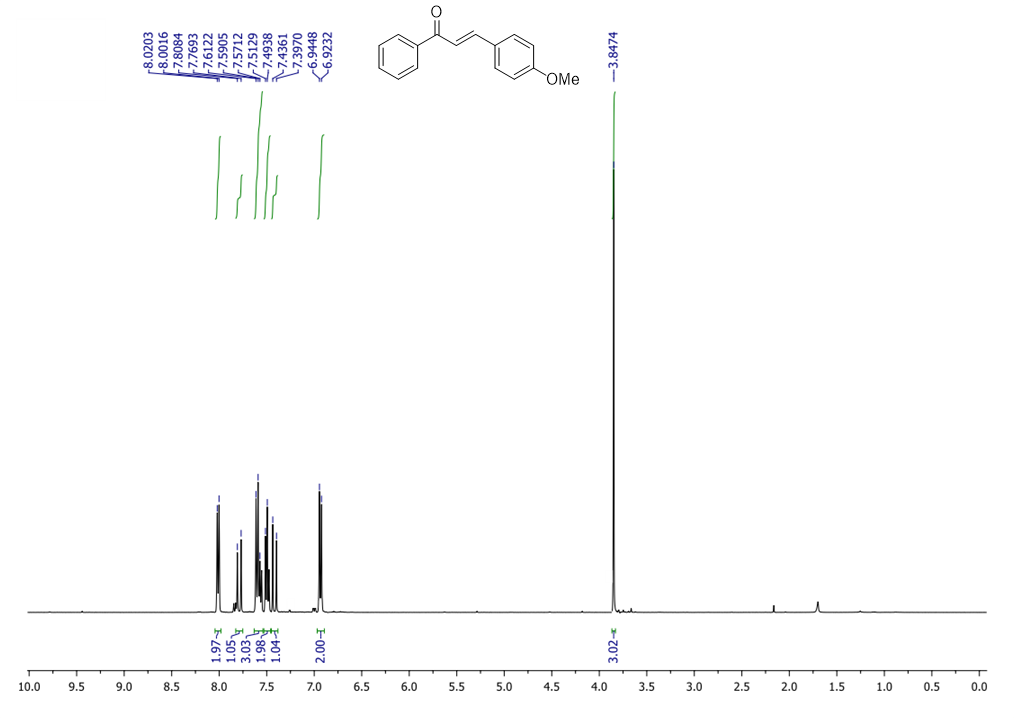


**Spectrum S11.** ^1^H-NMR of the compound (*E*)-3-(4-methoxyphenyl)-1-phenylprop-2-en-1-one (**068**).


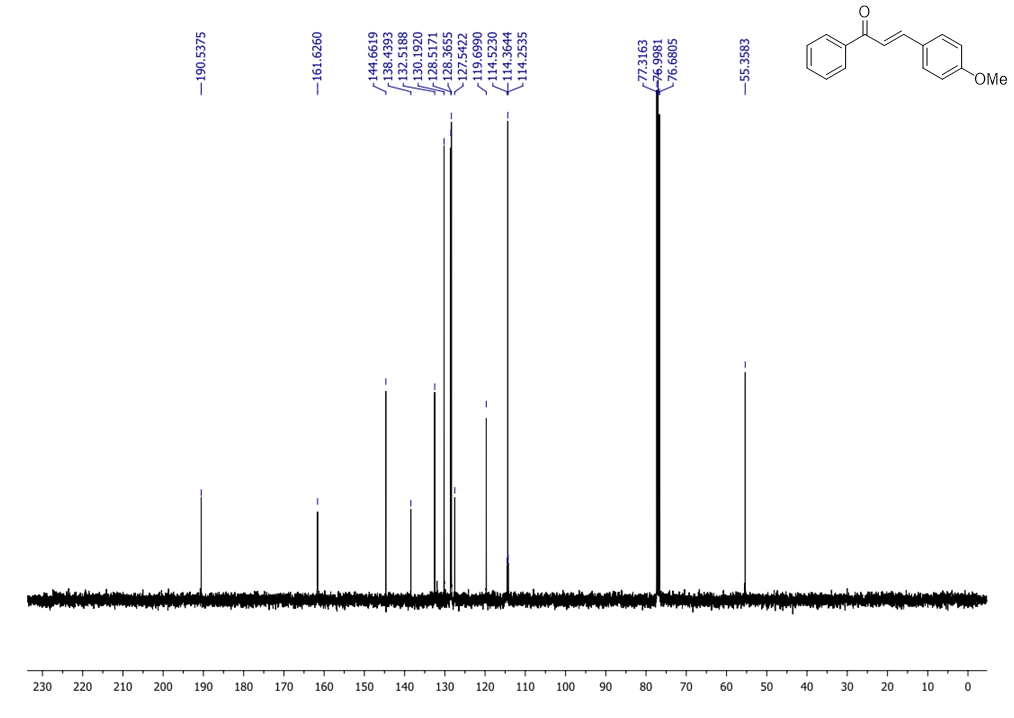


**Spectrum S12.** ^13^C-NMR of the compound (*E*)-3-(4-methoxyphenyl)-1-phenylprop-2-en-1-one (**068**).

**Spectrum S13.** FT-IR of the compound (*E*)-3-(4-hydroxy-3-methoxyphenyl)-1-phenylprop-2-en-1-one (**069**).


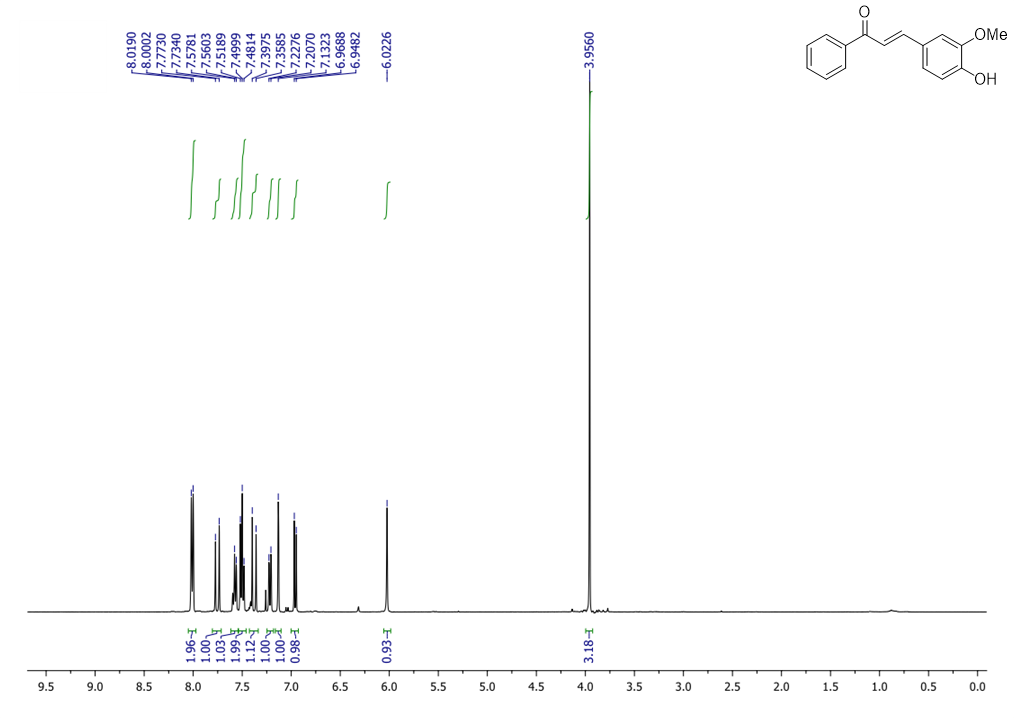


**Spectrum S14.** ^1^H-NMR of the compound (*E*)-3-(4-hydroxy-3-methoxyphenyl)-1-phenylprop-2-en-1-one (**069**).


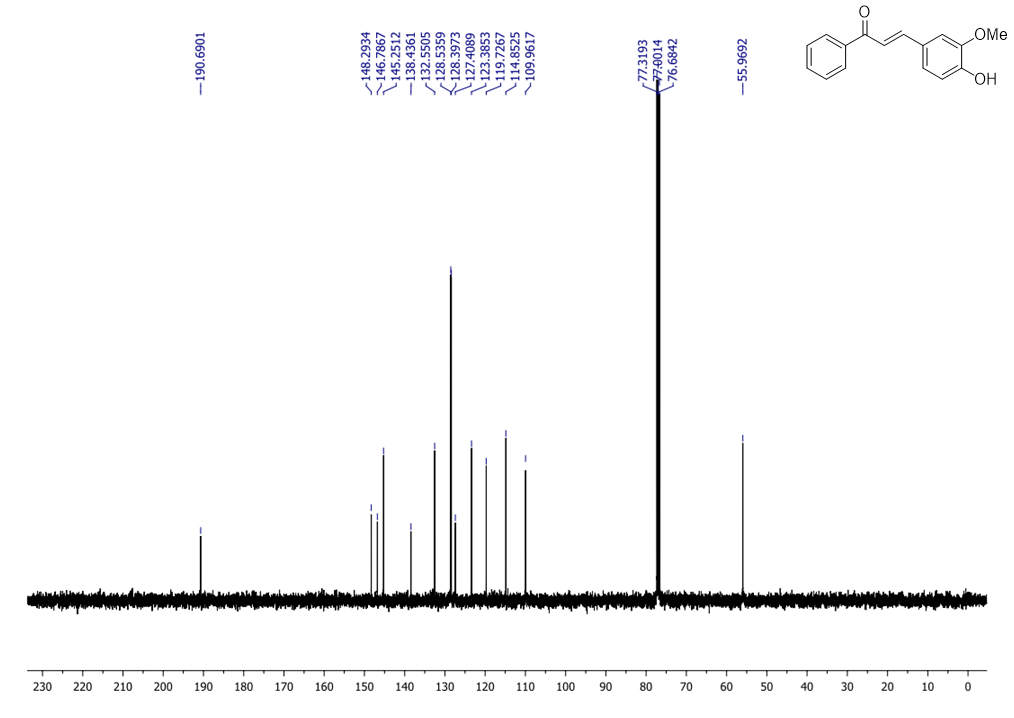


**Spectrum S15.** ^13^C-NMR of the compound (*E*)-3-(4-hydroxy-3-methoxyphenyl)-1-phenylprop-2-en-1-one (**069**).

**Spectrum S16.** FT-IR of the compound (*E*)-3-(3,4-dimethoxyphenyl)-1-phenylprop-2-en-1-one (**070**).


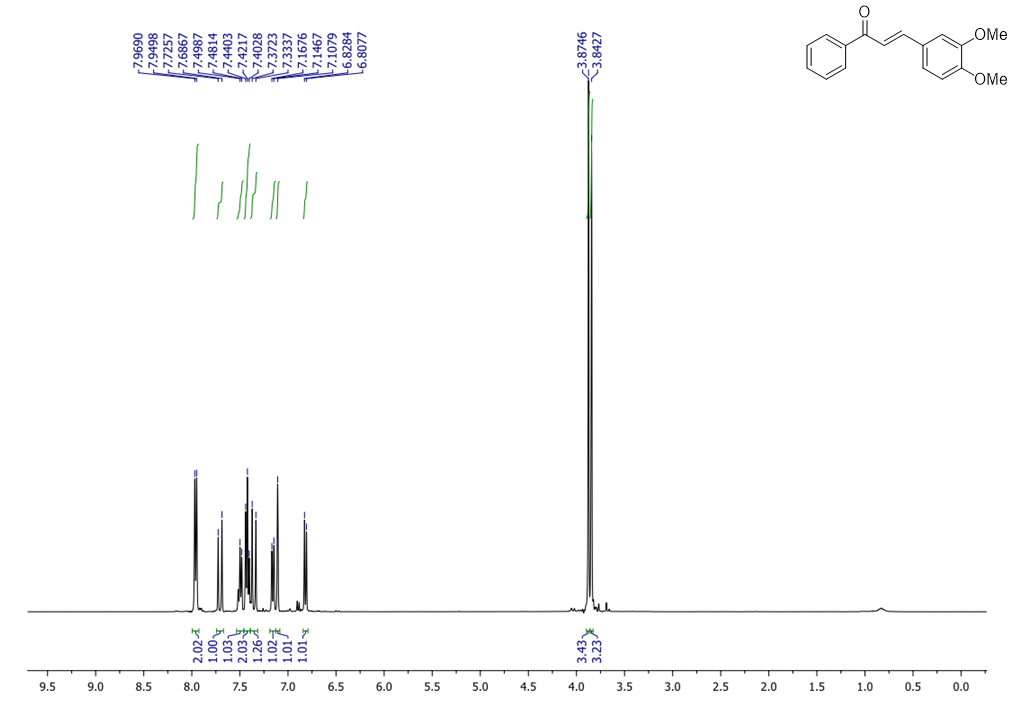


**Spectrum S17.** ^1^H-NMR of the compound (*E*)-3-(3,4-dimethoxyphenyl)-1-phenylprop-2-en-1-one (**070**).


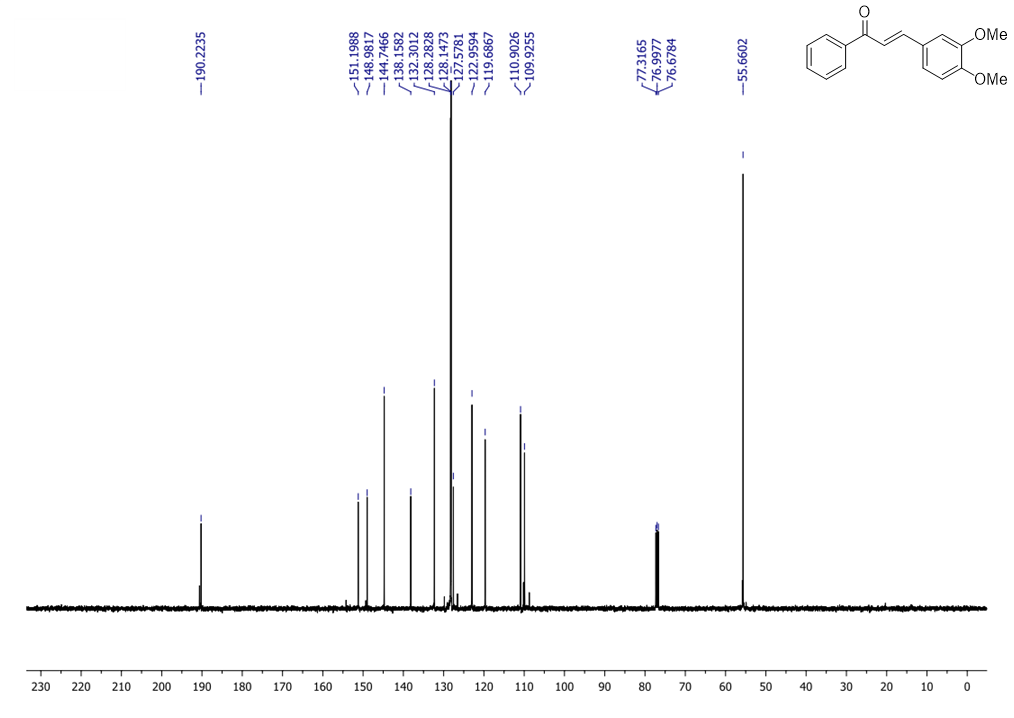


**Spectrum S18.** ^13^C-NMR of the compound (*E*)-3-(3,4-dimethoxyphenyl)-1-phenylprop-2-en-1-one (**070**).

**Spectrum S19.** FT-IR of the compound (*E*)-3-(benzo[*d*][1,3]dioxol-5-yl)-1-phenylprop-2-en-1-one (**071**).


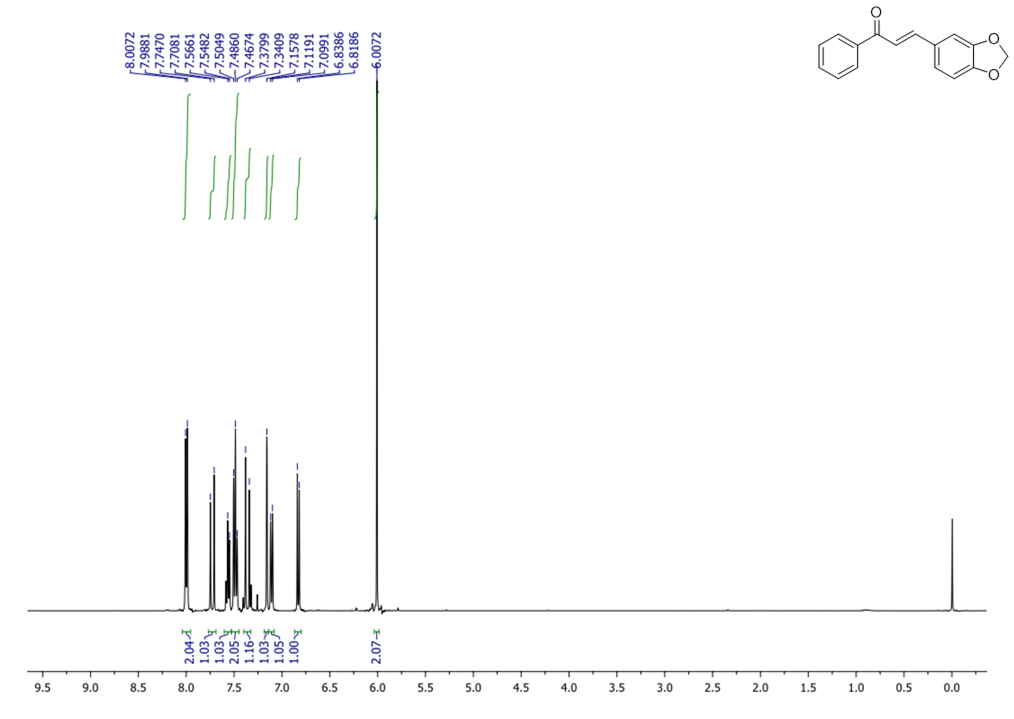


**Spectrum S20.** ^1^H-NMR of the compound (*E*)-3-(benzo[*d*][1,3]dioxol-5-yl)-1-phenylprop-2-en-1-one (**071**).


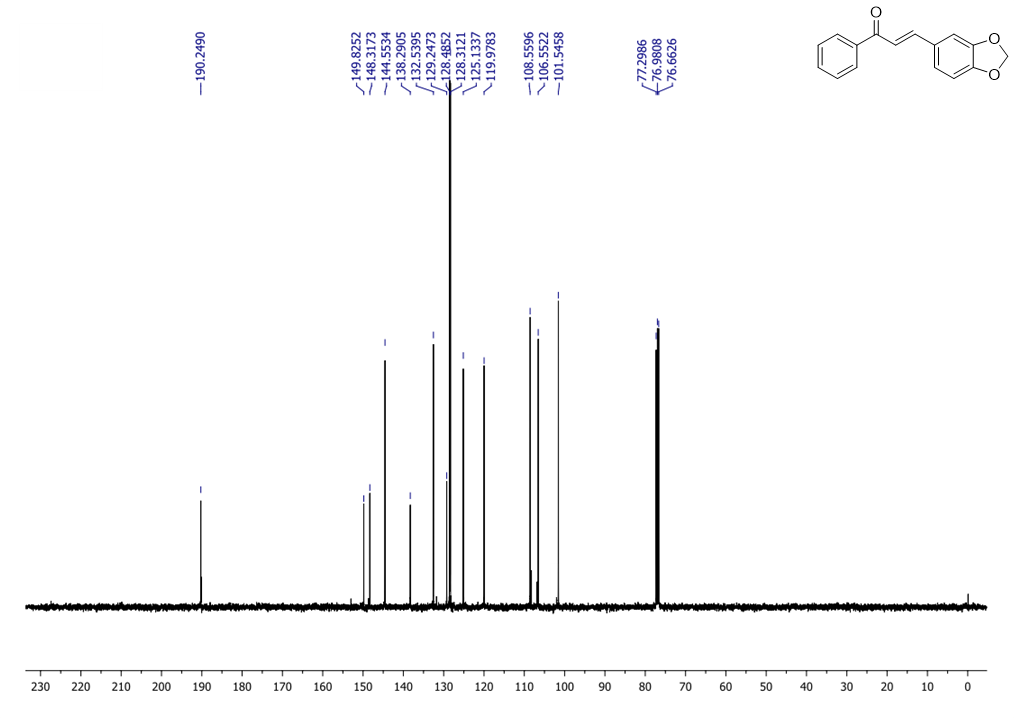


**Spectrum S21.** ^13^C-NMR of the compound (*E*)-3-(benzo[*d*][1,3]dioxol-5-yl)-1-phenylprop-2-en-1-one (**071**).

**Spectrum S22.** FT-IR of the compound (*E*)-1-phenyl-3-(3,4,5-trimethoxyphenyl)prop-2-en-1-one (**072**).


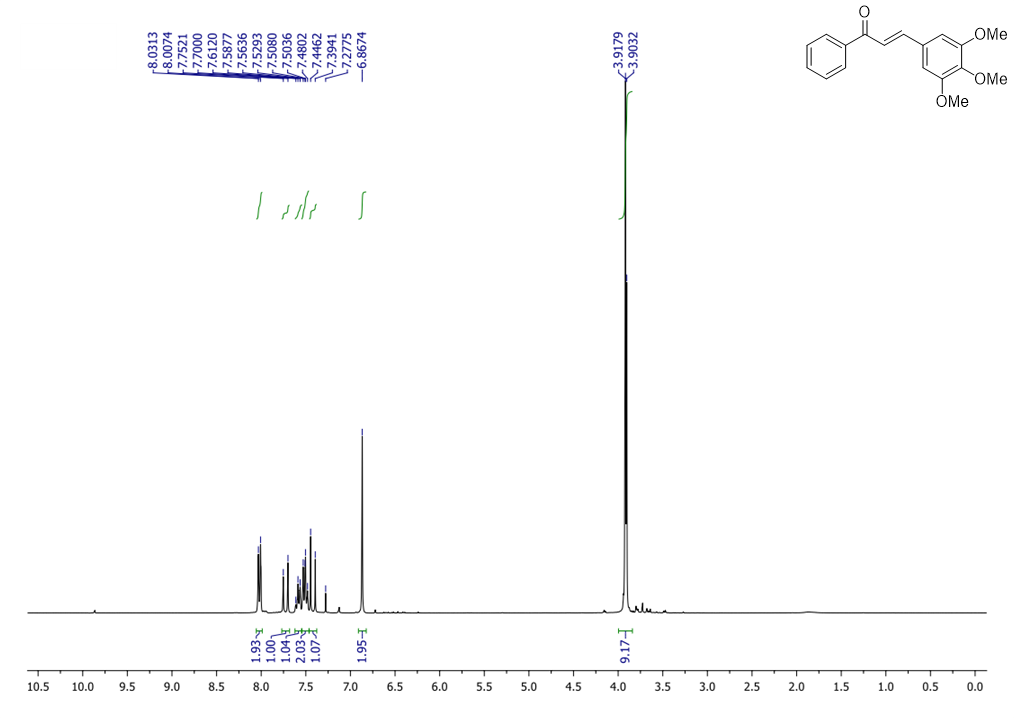


**Spectrum S23.** ^1^H-NMR of the compound (*E*)-1-phenyl-3-(3,4,5-trimethoxyphenyl)prop-2-en-1-one (**072**).


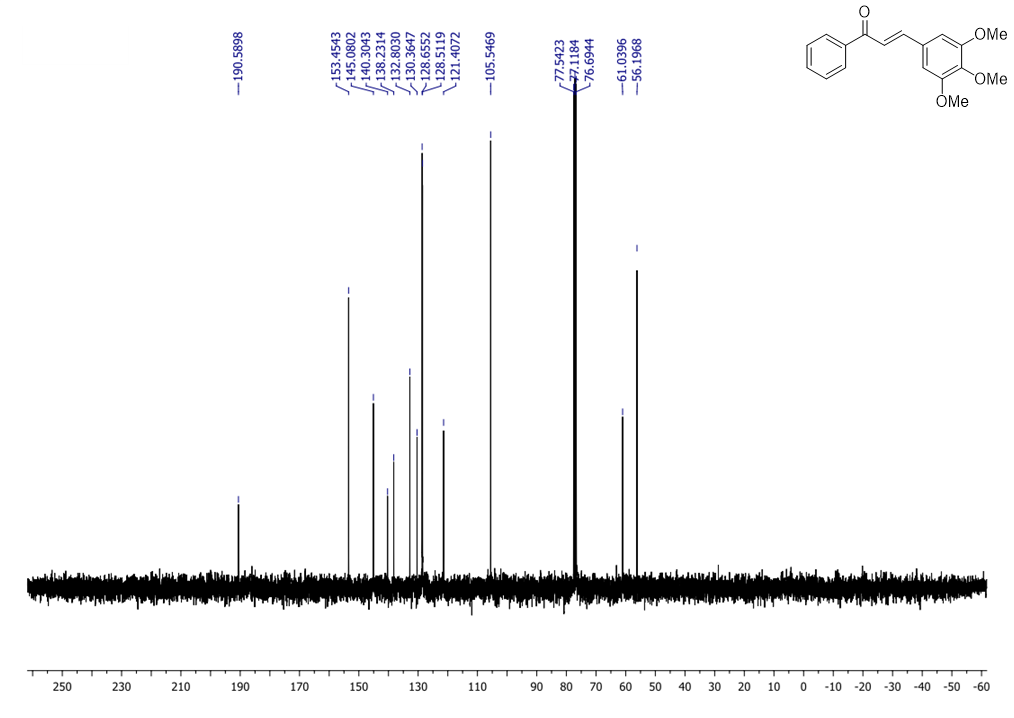


**Spectrum S24.** ^13^C-NMR of the compound (*E*)-1-phenyl-3-(3,4,5-trimethoxyphenyl)prop-2-en-1-one (**072**).

**Spectrum S25.** FT-IR of the compound (*E*)-3-(4-fluorophenyl)-1-phenylprop-2-en-1-one (**073**)


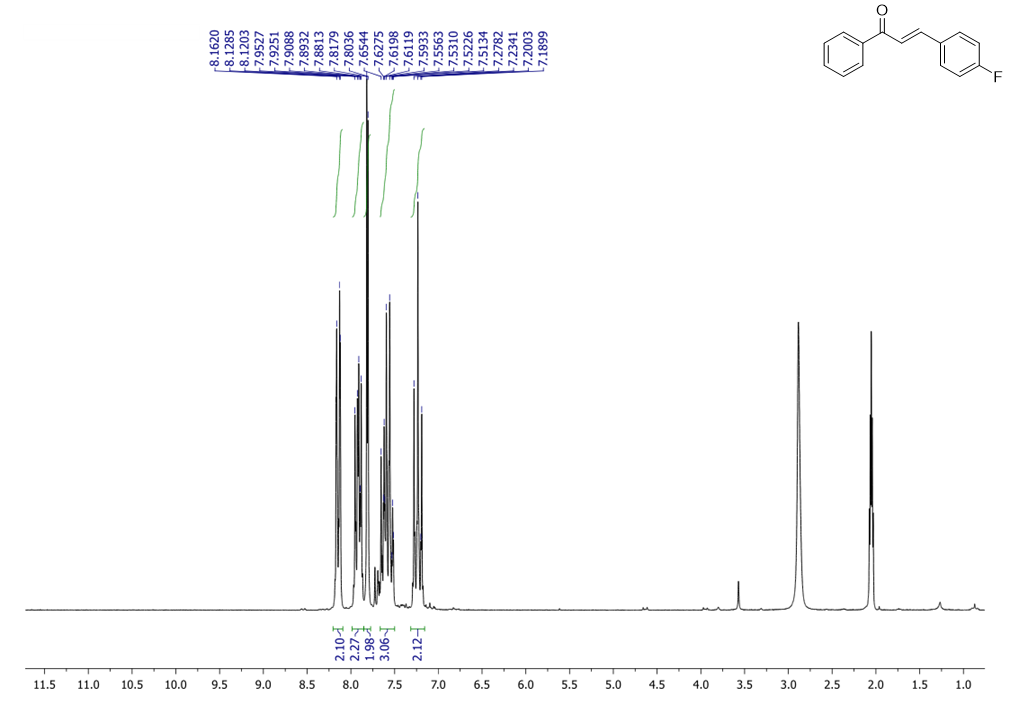


**Spectrum S26.** ^1^H-NMR of the compound (*E*)-3-(4-fluorophenyl)-1-phenylprop-2-en-1-one (**073**).


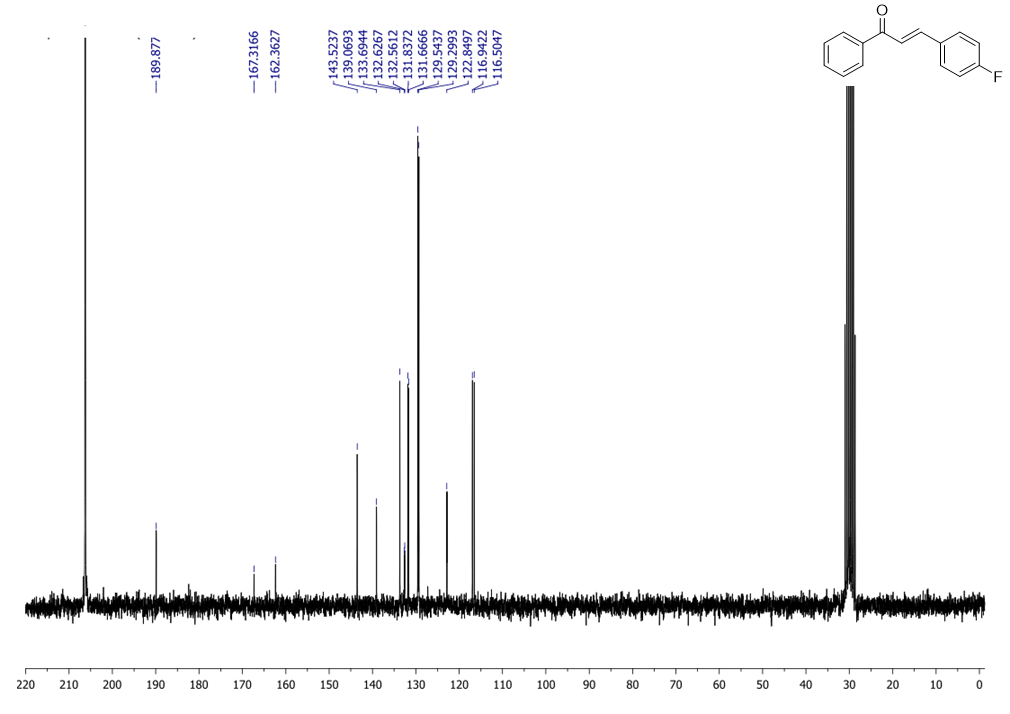


**Spectrum S27.** ^13^C-NMR of the compound (*E*)-3-(4-fluorophenyl)-1-phenylprop-2-en-1-one (**073**).

**Spectrum S28.** FT-IR of the compound (*E*)-3-(3-chlorophenyl)-1-phenylprop-2-en-1-one (**074**).


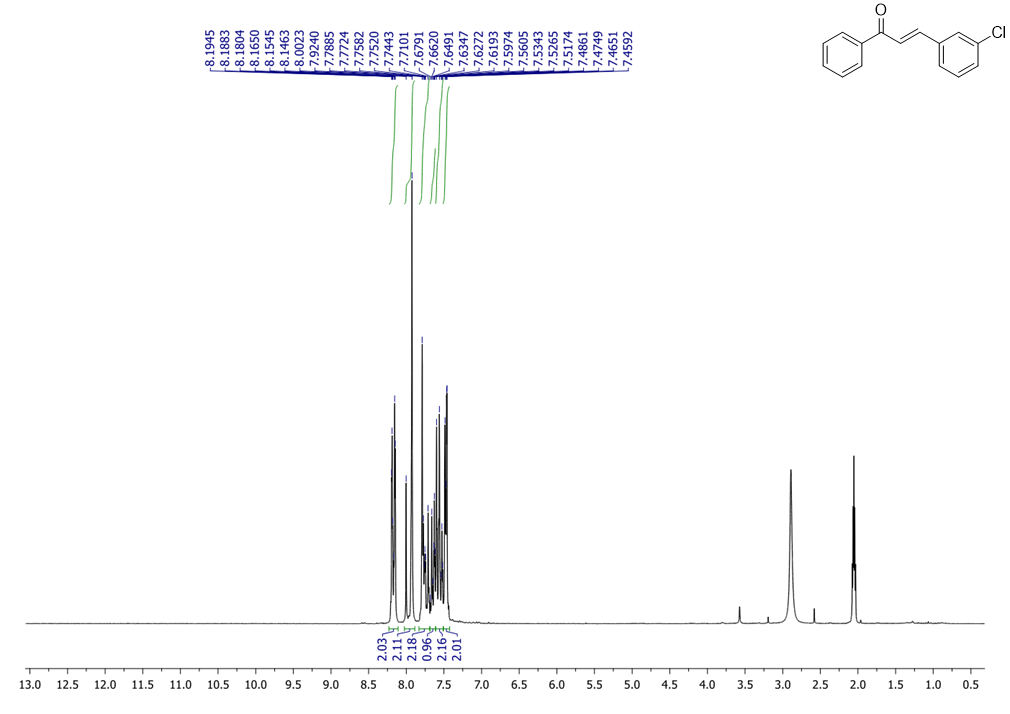


**Spectrum S29.** ^1^H-NMR of the compound (*E*)-3-(3-chlorophenyl)-1-phenylprop-2-en-1-one (**074**).


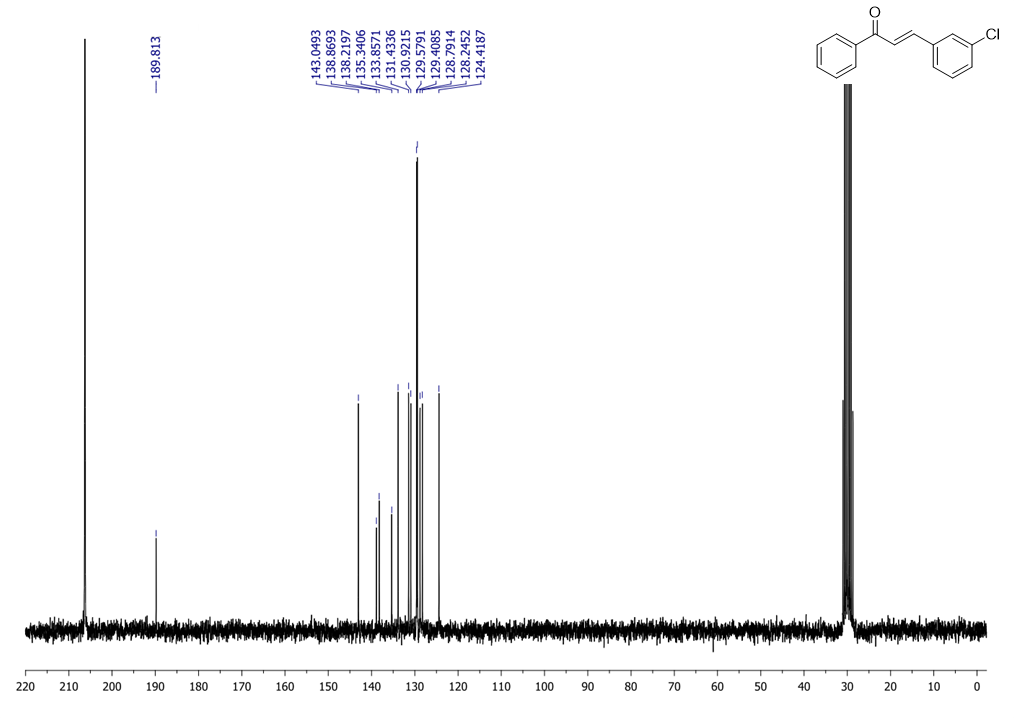


**Spectrum S30.** ^13^C-NMR of the compound (*E*)-3-(3-chlorophenyl)-1-phenylprop-2-en-1-one (**074**).

**Spectrum S31.** FT-IR of the compound (*E*)-3-(4-chlorophenyl)-1-phenylprop-2-en-1-one (**075**).


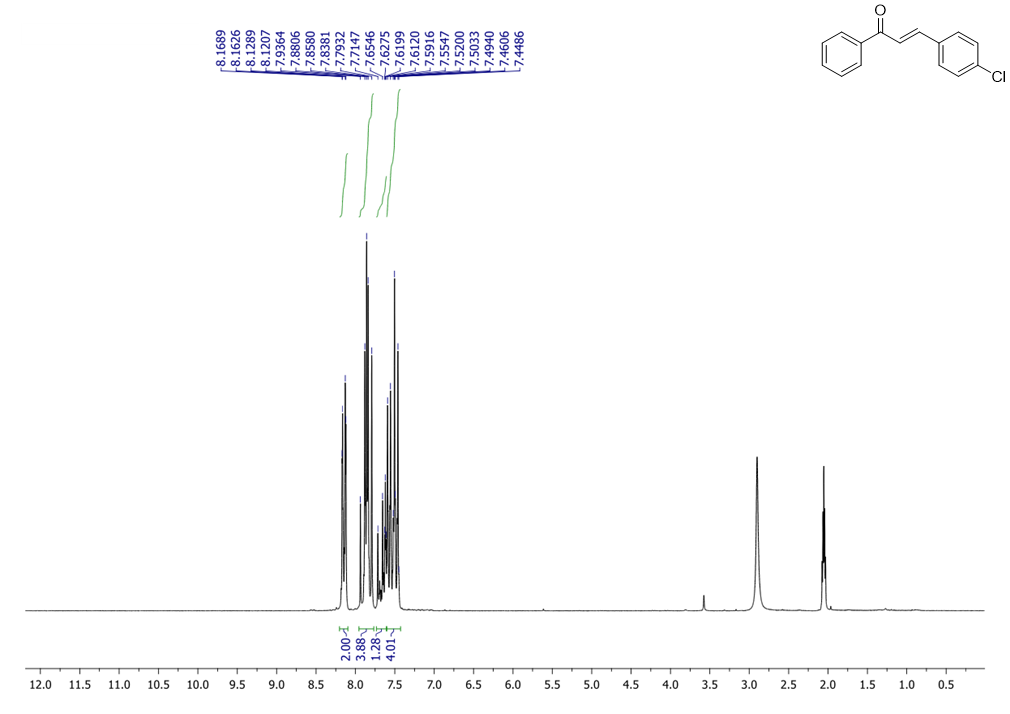


**Spectrum S32.** ^1^H-NMR of the compound (*E*)-3-(4-chlorophenyl)-1-phenylprop-2-en-1-one (**075**).


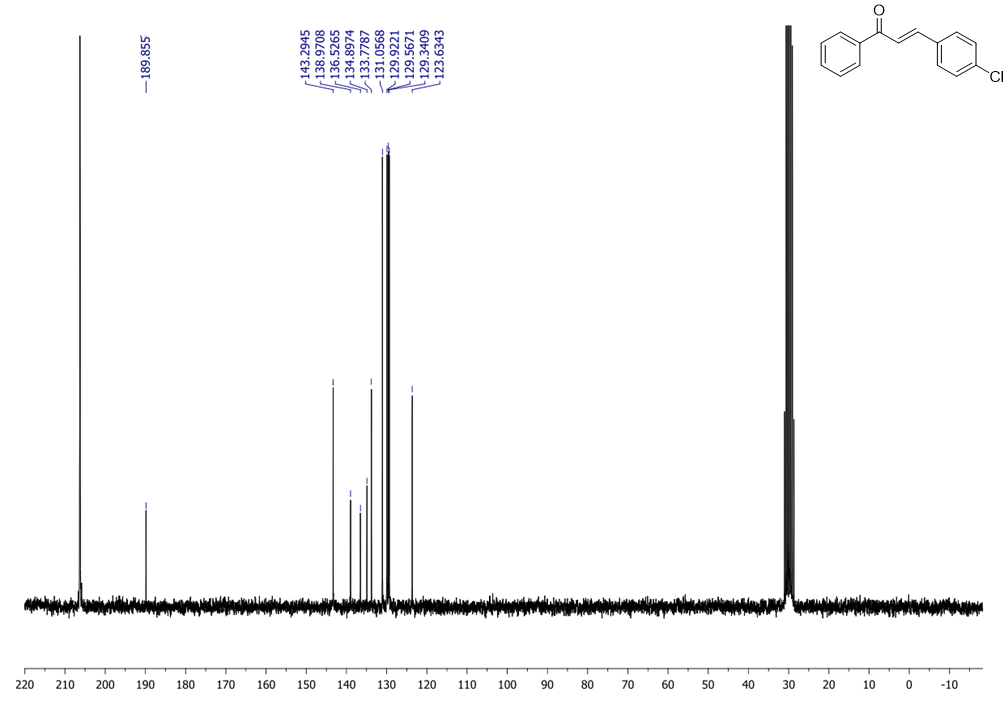


**Spectrum S33.** ^13^C-NMR of the compound (*E*)-3-(4-chlorophenyl)-1-phenylprop-2-en-1-one (**075**).

**Spectrum S34.** FT-IR of the compound (*E*)-3-(4-hydroxy-3-nitrophenyl)-1-phenylprop-2-en-1-one (**076**).


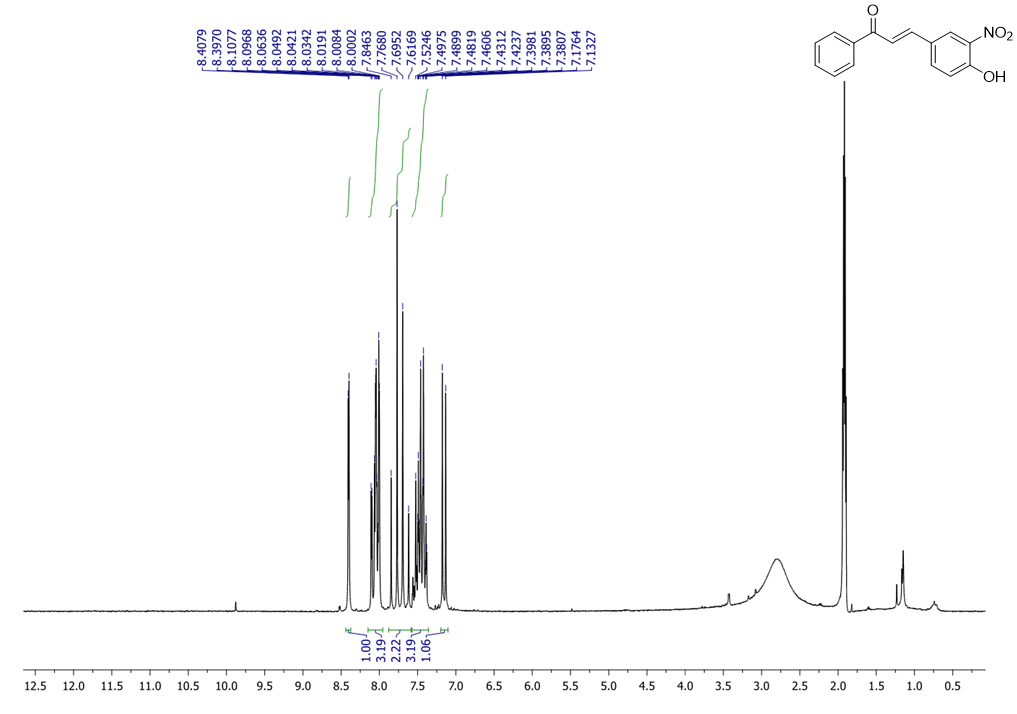


**Spectrum S35.** ^1^H-NMR of the compound (*E*)-3-(4-hydroxy-3-nitrophenyl)-1-phenylprop-2-en-1-one (**076**).


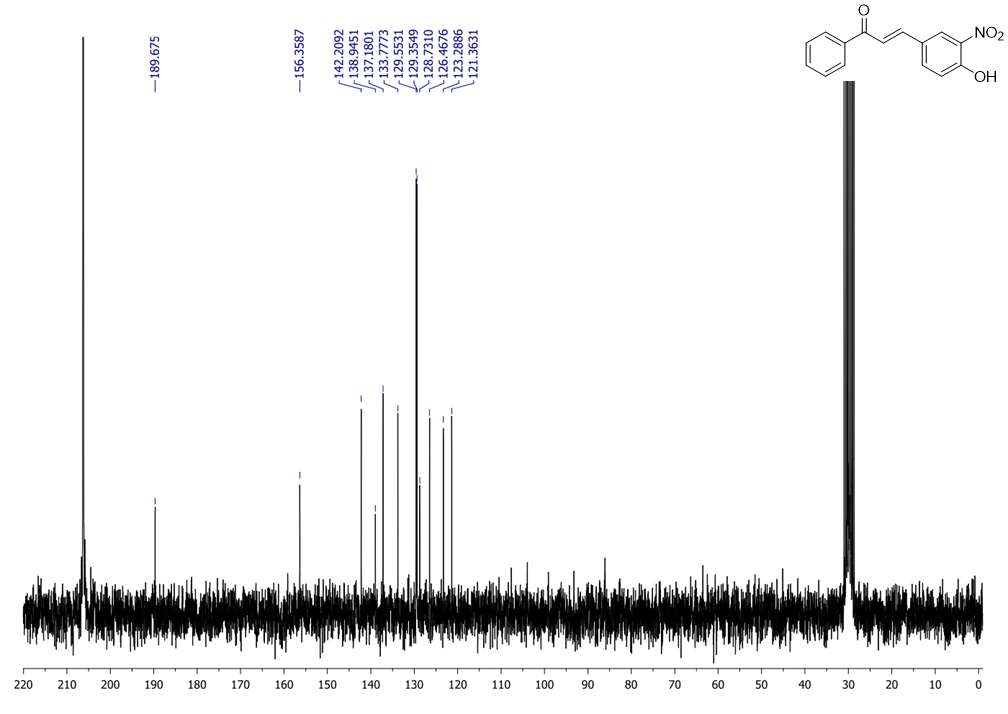


**Spectrum S36.** ^13^C-NMR of the compound (*E*)-3-(4-hydroxy-3-nitrophenyl)-1-phenylprop-2-en-1-one (**076**).

**3. Original images of biological assessment**


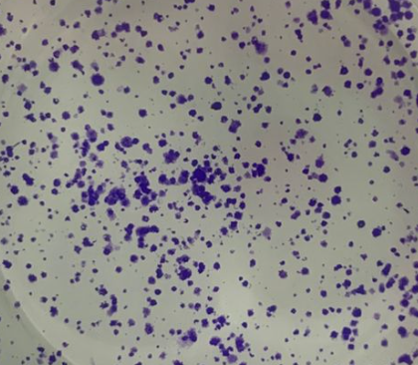


**Figure S3.** Original Image Figure 10B Compound 065.


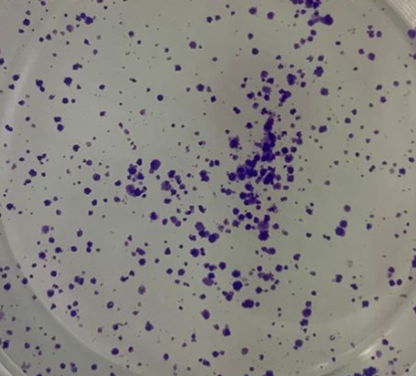


**Figure S4.** Original Image Figure 10B Compound 066.


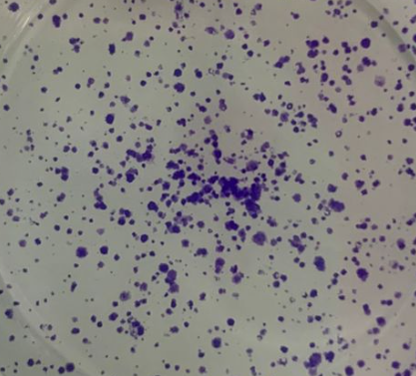


**Figure S5.** Original Image Figure 10B Compound 071.


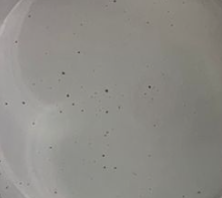


**Figure S6.** Original Image Figure 10B Compound 072.


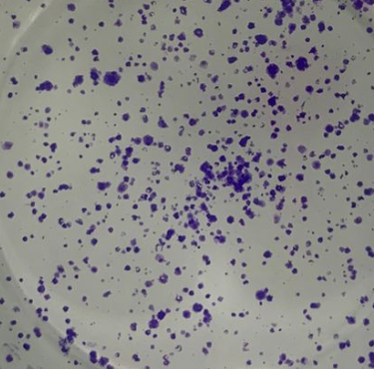


**Figure S7.** Original Image Figure 10B Compound 074.


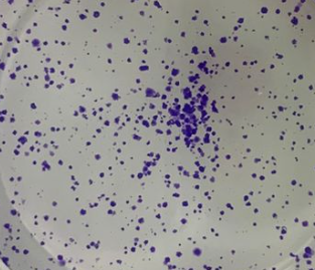


**Figure S8.** Original Image Figure 10B Control.


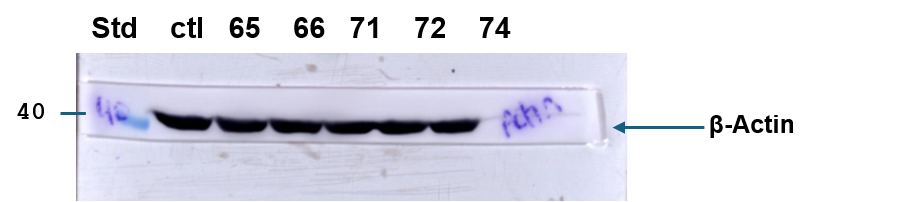


**Figure S9.** Original Image Figure 10D β-Actin.


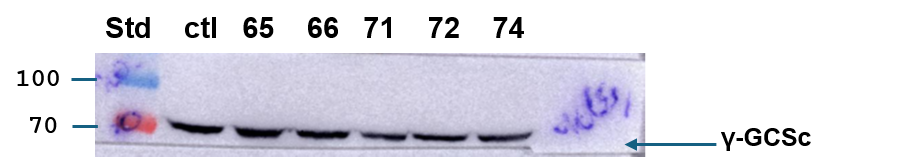


**Figure S10.** Original Image Figure 10D γ-GCSc.


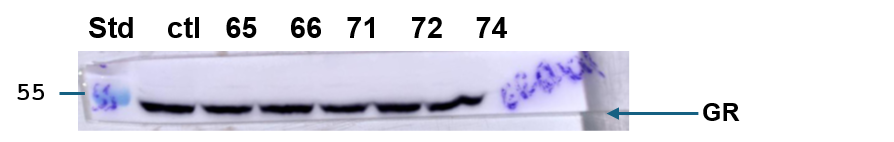


**Figure S11.** Original Image Figure 10D GR.


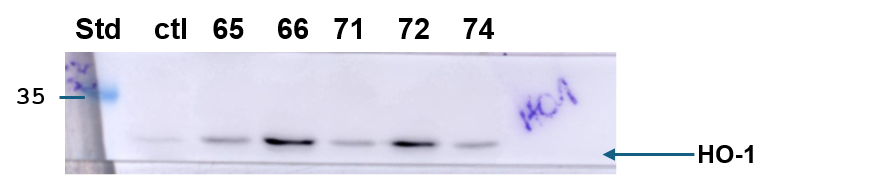


**Figure S12.** Original Image Figure10 D HO-1.


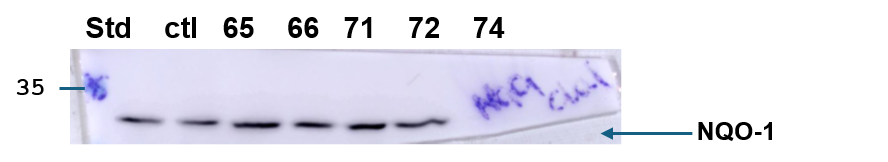


**Figure S13.** Original Image Figure 10D NQO-1.


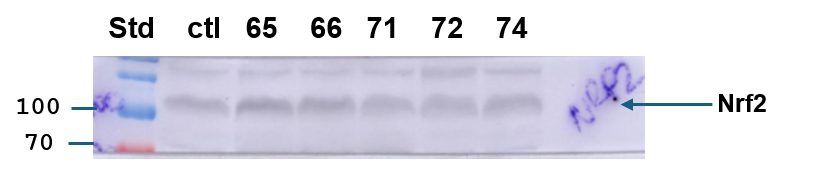


**Figure S14.** Original Image Figure 10D Nfr2.


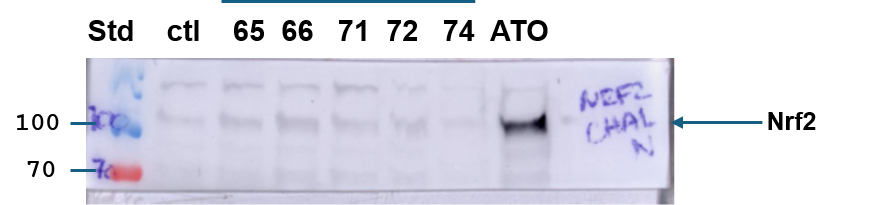


**Figure S15.** Original Image Figure 10E.
